# A genome-wide association study of radiotherapy induced toxicity in head and neck cancer patients identifies a susceptibility locus associated with mucositis

**DOI:** 10.1038/s41416-021-01670-w

**Published:** 2022-01-17

**Authors:** Line M. H. Schack, Elnaz Naderi, Laura Fachal, Leila Dorling, Craig Luccarini, Alison M. Dunning, The Head and Neck Group of the Radiogenomics Consortium, Gill Barnett, Miguel Elías Aguado Barrera, Neil G. Burnet, Laura M. Calvo, Brenda Diergaarde, Tom Dudding, Alison Dunning, Fréderic Duprez, Sarah L. Kerns, Melvin C. L. Kiang, Hans Langendijk, Hisham Mehanna, Andy Ness, Adelene Y. L. Sim, An Spiessens, Holly R. Summersgill, Juan F. Tajes, Ana Vega, Ceilidh Welsh, Enya O. H. Wen, Catharine West, The Danish Head and Neck Cancer Group (DAHANCA), Enya H. W. Ong, Melvin L. K. Chua, Johannes A. Langendijk, Behrooz Z. Alizadeh, Jens Overgaard, Jesper Grau Eriksen, Christian Nicolaj Andreassen, Jan Alsner

**Affiliations:** 1 Department of Experimental Clinical Oncology, Aarhus University Hospital, Aarhus, Denmark; 2 Department of Oncology, Aarhus University Hospital, Aarhus, Denmark; 3 University of Groningen, University Medical Center Groningen, Department of Radiation Oncology, Groningen, The Netherlands; 4 University of Groningen, University Medical Center Groningen, Department of Epidemiology, Groningen, The Netherlands; 5 Centre for Cancer Genetic Epidemiology, Department of Oncology, University of Cambridge, Cambridge, UK; 6 Wellcome Sanger Institute, Wellcome Genome Campus, Hinxton, UK; 7 Centre for Cancer Genetic Epidemiology, Department of Public Health and Primary Care, University of Cambridge, Cambridge, UK; 8 Division of Medical Sciences, National Cancer Centre Singapore, Singapore, Singapore; 9 Division of Radiation Oncology, National Cancer Centre Singapore, Singapore, Singapore; 10 Oncology Academic Clinical Programme, Duke-NUS Medical School, Singapore, Singapore; 11 Oncology Centre, Addenbrooke’s Hospital, Cambridge University Hospitals NHS Foundation Trust, Cambridge, UK; 12 Genetic Unit, Institute of Legal Medicine and Genomic Medicine Group, USC Faculty of Medicine, Madrid, Spain; 13 The Christie, Department of Radiation Oncology, Division of Cancer Sciences, University of Manchester, Manchester, UK; 14 Graduate School of Public Health; UPMC Hillman Cancer Center, University of Pittsburgh, Pittsburgh, PA USA; 15 MRC Integrative Epidemiology Unit, Department of Population Health Sciences, Bristol Medical School, University of Bristol, UK AND Bristol Dental School, University of Bristol, Bristol, UK; 16 Centre for Cancer Genetic Epidemiology, University of Cambridge, Strangeways Research Laboratory, Cambridge, UK; 17 Department of Radiation Oncology, Ghent University Hospital and Ghent University, Ghent, Belgium; 18 Wilmot Cancer Institute, Department of Radiation Oncology, University of Rochester Medical Center, Rochester, NY USA; 19 Division of Radiation Oncology, NCCS—National Cancer Centre Singapore, Singapore, Singapore; 20 Institute of Head and Neck Studies and Education, Institute of Cancer and Genomic Sciences, University of Birmingham, Birmingham, UK; 21 NIHR Bristol Biomedical Research Centre, University of Bristol NHS Foundation Trust and University of Bristol, Bristol, UK; 22 Division of Medical Sciences, NCCS—National Cancer Centre Singapore, Singapore, Singapore; 23 Department of Radiation Oncology and Experimental Cancer Research, Ghent University Hospital, Ghent, Belgium; 24 Manchester Cancer Research Centre, Faculty of Life Sciences and The Christie NHS Foundation, Trust, University of Manchester, Manchester, UK; 25 Department of Oncology, University of Cambridge, Cambridge, UK; 26 Institute of Cancer Sciences, The University of Manchester, Manchester Academic Health Science Centre, Manchester, UK

**Keywords:** Head and neck cancer, Prognostic markers

## Abstract

**Purpose:**

A two-stage genome-wide association study was carried out in head and neck cancer (HNC) patients aiming to identify genetic variants associated with either specific radiotherapy-induced (RT) toxicity endpoints or a general proneness to develop toxicity after RT.

**Materials and methods:**

The analysis included 1780 HNC patients treated with primary RT for laryngeal or oro/hypopharyngeal cancers. In a non-hypothesis-driven explorative discovery study, associations were tested in 1183 patients treated within The Danish Head and Neck Cancer Group. Significant associations were later tested in an independent Dutch cohort of 597 HNC patients and if replicated, summary data obtained from discovery and replication studies were meta-analysed. Further validation of significantly replicated findings was pursued in an Asian cohort of 235 HNC patients with nasopharynx as the primary tumour site.

**Results:**

We found and replicated a significant association between a locus on chromosome 5 and mucositis with a pooled OR for rs1131769*C in meta-analysis = 1.95 (95% CI 1.48–2.41; *p*_pooled_ = 4.34 × 10^−16^).

**Conclusion:**

This first exploratory GWAS in European cohorts of HNC patients identified and replicated a risk locus for mucositis. A larger Meta-GWAS to identify further risk variants for RT-induced toxicity in HNC patients is warranted.

## Introduction

Head and neck cancers (HNC) represent a group of patients with increasing loco-regional control and survival rates [1–3]. Curative treatment is usually made up of surgery, radiotherapy (RT) or a combination. Primary RT is often preferred for reasons of cosmesis and preserved organ function. However, RT also comes with the price of RT-induced toxicity. In HNC this toxicity is primarily characterised by mucositis, dysphagia, fibrosis of the skin, atrophy of the epithelial linings and xerostomia. The risk of RT-induced toxicity is influenced by various treatment and patient-related factors. A review of normal tissue complication (NTCP) models from 2010–2017 in standard fractionated RT of HNC identified the following parameters to be associated with RT-induced toxicity endpoints: Mean dose, maximal dose, irradiated volumes, the dose leading to a 50% complication rate (*D*_50_), RT technique (Intensity-Modulated Radiation Therapy (IMRT) vs. 3D Conformal RT technique), gender, concomitant chemotherapy and stage of T-site [4]. However, increasing evidence also points to an individual inherent predisposition of developing toxicity [5] and in 2009, the Radiogenomics Consortium [6, 7] was established, facilitating research efforts in genetic variations associated with RT-induced toxicity. In studies of breast-, head and neck and prostate cancer cohorts, several SNPs associated with different morbidities after RT have been identified [8–11]. Two recent GWA studies explored RT-induced toxicity (temporal lobe brain injury and oral mucositis, respectively) in nasopharyngeal HNC patients [12, 13]. And another recent study of 37 patients identified associations between somatic genetic variants in HNC tumour tissue and severity of RT-induced morbidity, which is the first study to link genetic variants in the tumour to surrounding normal tissue morbidity [14]. However, GWASs with respect to radiation-induced toxicity in non-nasopharyngeal HNC patients remain to be investigated. The present study aimed to identify genetic variants associated with either specific RT-induced toxicity endpoints or a general proneness to develop toxicity after RT across endpoints. We conducted a two-stage genome-wide study comprising a total of 1780 European HNC patients with larynx and oro/hypopharynx as the primary tumour site. Further validation was pursued in an Asian cohort of HNC patients with nasopharynx as the primary tumour site.

## Materials and methods

We performed a two-stage GWAS including 1780 HNC patients with oropharyngeal/hypopharyngeal or laryngeal cancer from two European cohorts. In a discovery study including 1183 patients, common genetic variants were tested for their association with either specific radiation-induced morbidities or with a general susceptibility to develop toxicity after RT across endpoints. In a replication study including 597 patients, the genome-wide significant variants identified in the discovery study were tested in an independent cohort of comparable HNC patients and if successfully replicated, variants were meta-analysed. A subsequent attempt to replicate significant findings in a cohort of 235 Asian HNC patients with nasopharyngeal cancer was made.

### Discovery study

#### DAHANCA cohorts

Patients in the discovery study were treated within The Danish Head and Neck Cancer Group (DAHANCA) protocols in Denmark for head and neck squamous cell carcinoma in the period of 2000–2011. Eligibility criteria were stringently set to ensure a uniform cohort as follows: Histologically verified pharyngeal or laryngeal squamous cell carcinomas T1-4N0-3M0. Curative intent treatment regimen, consisting of primary RT ± concomitant treatment, >600 days recurrence-free (curative single lymph node extirpation on the neck was accepted) and available biological tissue with germline DNA. Patients were excluded by the death of any cause or switch to palliative treatment regimen during primary RT. Patients were censored by recurrence date in case of recurrence after 600 days. RT doses were 66, 68 (accelerated 6 fx/week regime) or 70 Gy (standard 5 fx/week regime). Fractionation doses were 2 Gy/fx. Concomitant treatment included weekly cisplatin and Nimorazole. Cisplatin was offered to patients with N1–3 disease from 2007, dosage 40 mg/m^2^ once weekly during RT. Nimorazole was administered to all patients except patients with glottic laryngeal cancers T1N0M0. RT technique was IMRT from 2006. Before this, 3D-Conformal RT was applied. The 1183 patients included were from the following cohorts:

DAHANCA10 (*N* = 81): In a randomised phase III study, the effect of Darbepoetin-alfa in anaemic patients (haemoglobin < 14.5 g/dl for men and < 13.5 g/dl for women) was analysed with loco-regional failure as the primary endpoint. Patients were eligible by the following criteria: Squamous cell carcinoma of the larynx, oropharynx, hypopharynx or oral cavity T1-4N0-3M0 (excluding stage I glottic carcinomas), WHO performance score 0–2, age ≥18 years. The study was interrupted after an interim analysis showing the inferiority of the Darbepoetin-alfa arm [15].

DAHANCA19 (*N* = 485): In a randomised, open-label phase III trial in 2007–2012, the effect of zalutumumab, an EGFR-inhibitor, was analysed with loco-regional failure as the primary endpoint. Patients were eligible by histologically verified squamous cell carcinoma of the oral cavity, larynx, oropharynx or hypopharynx, stage T1-4N0-3M0 (excluding stage I + II glottic carcinomas), WHO performance score 0–2, age ≥18 years [16].

DAHANCA Prospective biobank protocol (*N* = 617): Consecutive patients with available biobank material, age ≥ 18 years, treated according to standardised national DAHANCA guidelines [17].

#### Endpoints

Follow-up data were prospectively registered weekly during RT and after completion of RT course, at 2 weeks and at 3, 6, 12, 24, 36, 48 and 60 months. RT-induced toxicity in the present study was scored as the maximal grade at any time during treatment or follow-up.

Acute toxicity endpoints comprised acute dysphagia and mucositis between week 2 after RT start and 14 days after treatment completion. Late endpoints comprised late dysphagia, xerostomia, mucosal atrophy and skin fibrosis between 600 days and 5 years after treatment completion. Under the hypothesis that mucosal atrophy and skin fibrosis are manifestations of similar biological pathway processes, these entered a combined fibrosis/atrophy endpoint. Additionally, tube feeding at 6 months was included as a binary variable. This endpoint represents a composite multifactorial endpoint dependent on not only RT- and patient-related factors but also on factors such as inter-institutional variation in the availability of specialised knowledge in tube feeding and cultural differences in when to start and when to end tube feeding. It was included as a surrogate marker for severe toxicity after RT.

Acute and late endpoints were registered ordinally according to standardised DAHANCA forms, which are comparable with LENT-SOMA scales [18]. For the purpose of the present study, endpoints were dichotomised into “moderate-severe” and “severe”. Grading and cut-off points are available in Supplementary Table 1. Severe mucositis (corresponding to ulceration) was not tested independently due to a low number of events.

With the purpose of normalising and combining acute, late and global endpoints, Standardised Total Average Toxicity (STAT) scores were estimated aggregating ordinal endpoints (Supplementary Table 1). This method was previously described elsewhere [19]. STAT_acute_ included acute dysphagia and mucositis. STAT_late_ included late dysphagia, xerostomia, fibrosis and atrophy. STAT_global_ combined STAT_late_ and STAT_acute_. Tube feeding was not included in the STAT scores.

#### Covariates

Available covariates were age, sex, total RT dose, concomitant chemotherapy, protocol, and a surrogate for irradiated volume (Supplementary Table 1). As dose-volume histograms were not available, a trichotomised surrogate marker for irradiated volume was estimated from the cancer site and TNM stage. The rationale was as follows: Irradiated volumes depended on site and stage. Patients with T1a-T1bN0M0 glottic laryngeal carcinomas received small box fields to the larynx without an elective volume. The remaining patients with N0 disease received bilateral (or unilateral for tonsillar tumours without the involvement of soft palate or base of tong) elective volumes by the following criteria: Oropharyngeal and laryngeal cancers without subglottic disease: lymph node levels II and III. Hypopharyngeal cancers: levels II, III and IV. Subglottic laryngeal cancers: levels II, III, IV and VI. In general, patients with N1–3 disease received a larger irradiated volume than patients with N0 disease as the positive nodes were irradiated to the prescribed tumour dose and elective volumes could be expanded beyond those described above. Based on this, the volume surrogate cut-offs were: Volume 1 = T1a-1bN0M0 glottic laryngeal carcinomas, Volume 2 = all other TxN0 sites, Volume 3 = TxN1-3 carcinomas.

#### Genotyping, imputation, quality control and bioinformatics

Germline whole-genome DNA was extracted from buffy coats and SNP genotyping was done using the Infinium OncoArray-500K Bead Chip (Illumina Inc., San Diego, USA). Quality control processes adhered to OncoArray guidelines [20] and are available in Supplementary Document and Supplementary Table 2. Haplotype estimation (phasing) was done using SHAPEITv2(r837). Imputation was done with IMPUTE2 using the last phase of 1000 Genomes, mapped to build GRCh37/hg19 as a reference.

#### Statistical analysis

The cohort was analysed as a pooled dataset of 1183 patients from DAHANCA10, DAHANCA19 and the DAHANCA Prospective biobank protocol as they were eligible by the criteria set. Covariates considered clinically relevant were tested against endpoints in a univariate logistic regression model for binary endpoints and a linear regression model for STAT (continuous) scores (Supplementary Fig. 1). If significant at an α = 0.05 level they entered multivariate analysis and by the method of backwards stepwise selection, final models were created. The total dose was included in the final models for all endpoints. The volume surrogate entered models for all endpoints except severe late dysphagia and tube feeding. Concomitant chemotherapy entered models for acute endpoints. Sex entered models for xerostomia and STAT scores. The protocol entered models for xerostomia, STATlate and STATglobal. Principal components were introduced stepwise in analysis and included if significant (α = 0.05).

Associations between genotypes and endpoints were tested using a logistic regression model for binary endpoints and a linear regression model for STAT (continuous) scores. All analyses were adjusted for covariates in a case-control design. A log-additive genetic model was assumed for single endpoints and a linear model was assumed for STAT scores. SNPs were included as a number of effect alleles or imputed genotype dosage resulting in a per-allele odds ratio (OR) or a regression coefficient (β) for each SNP. The significance level was set to *p* < 5 × 10^−8^ and thus was not adjusted for testing multiple endpoints. This choice was based on including genetic variants suggestively significant in the replication phase.

In a Bayesian approach, we estimated the posterior probability of a false discovery (p_Bayes_), assuming a prior probability = 10^−4^ and a prior variance = 0.35^2^ [21, 22]. Top variants with a posterior probability <10% were considered a plausible true positive discovery. Data preparation and statistical analyses were carried out using Stata13 (StataCorp, TX USA), R Statistical Package v3.3.0 [23] and SNPTEST [24]. The study is reported in compliance with the STROGAR guidelines for radiogenomic studies [25].

#### Power analysis

Power was estimated using the Genetic Association Study (GAS) Power Calculator [26] and is illustrated for selected endpoint frequencies in Supplementary Fig. 2. For the discovery phase, in general associations with ORs <1.7 were not likely to be identified with a power of 80%.

### Replication studies

#### Dutch cohort

The study population consisted of 1102 HNC patients eligible by the same criteria as in the discovery study and treated with definitive or postoperative RT or Chemo-RT from 2007 to 2017. All patients were included in the UMCG-HNC prospective data registration program, in which all baseline patient-, tumour- and treatment characteristics, as well as acute and late toxicity, were prospectively scored. Patients were followed up to 5 years after completion of treatment (NCT02435576 at clinicaltrials.gov).

#### Endpoints, SNPs and covariates

A replication study was performed on genetic variants reaching GWAS significance in the discovery phase and including suggestively significant associations with *p* < 10^−5^ for the specific endpoint. The replication study included the same covariates associated with the endpoint as in the discovery study. In the discovery cohort, acute toxicity was assessed weekly up to the second week after completion of treatment, whereas in the replication cohort, acute toxicity was assessed weekly during radiation and up to 5 weeks after completion of RT. The included endpoint, mucositis, was defined as moderate or severe on at least one time point between the second week after the start of RT and the fifth week after completion of RT.

#### Genotyping, imputation, quality control and bioinformatics

Out of 1,102 patients, 607 were genotyped on Illumina human hap 550k v.3.0 and 495 on Illumina global screening array. Quality control procedures were performed using standard procedures. First, samples and SNPs with call rates below 98% and MAF < 1% were removed. We checked the ethnicity of subjects using multidimensional scaling (MDS) clustering of our samples with HapMap Phase3 individuals using EIGENSTRAT [27]. Samples that deviated more than 3 SD from the mean of their closest clusters were removed. Second, genotypes that passed the QC were imputed on the backbone of Haplotype Reference Consortium reference panel (HRC) version R1.1. Third, we further performed post-imputation QC involved removing SNPs with an imputation quality (info) score of R^2^ < 0.3, with a MAF < 0.01, SNPs that had a discordant MAF (maximum allowed difference < 0.15) compared to the reference panel, and strand ambiguous AT/CG SNPs and multi-allelic SNPs. Two imputed datasets were then merged together using PLINK (version 1.90). As the genotyping array differed between the discovery and the replication cohorts, some variants from the discovery phase were not reproduced and thus not analysed in the replication phase.

#### Statistical analysis

We used logistic regression analysis and included covariates similar to the discovery study. The top ten principal components were included. The effect estimates were reported as OR with their corresponding 95% CI and associated *p*-values. The summary results of both discovery and replication studies were meta-analysed using an inverse variance-weighted fixed effects model as implemented in METAL (version 2011-03-25) [28]. A *p*-value <0.05 was considered statistically significant for replication analysis. For the meta-analysis, we maintained a significance threshold of *p* < 5 × 10^−8^. In all cases, it was a part of the quality control to check that directionality of effect and that MAFs were comparable between discovery and replication phases.

#### Singapore cohort

Genetic variants identified in the discovery phase and successfully replicated were independently tested in an Asian cohort of 235 HNC patients with nasopharyngeal carcinoma treated with curative intent with Radiotherapy (70 Gy/35 fractions) ± chemotherapy (clinicaltrials.gov: NCT04340024). Germline DNA was whole-exome sequenced using the Agilent V6 + UTR NovaSeq platform. Genotyping, quality control and bioinformatics was performed using Genome Analysis Tool KIT (GATK v 4.1.8.0) and adhering to the GATK best practices workflow (https://github.com/gatk-workflows/gatk4-germline-snps-indels). Germline variants were called for each sample using HaplotypeCaller [29]. This was followed by a joint genotyping step via GATK GenotypeGVCF across the whole cohort. The cohort followed the same protocol as the Dutch cohort in terms of endpoints, covariates included, statistical analysis methods and *p*-value significance cut-off.

## Results

### Discovery study

#### Genotyping and patient characteristics

Genotyping and imputation resulted in a dataset of 14,640,905 common variants in 1237 patients including duplicate controls. After Quality Control processes, 7,178,424 variants (SNPs, deletion/insertion variations (indels) and copy number variations (CNVs)) and 1183 patients remained in analysis. Stepwise outcomes from QC processes are available in Supplementary Table 2. Clinical characteristics are displayed in Table 1.

**Table 1:** Patient characteristics of discovery and replication cohorts.

	Discovery study				Replication studies	
	All patients *N* (%)	DAHANCA Prosp. biobank protocol *N* (%)	DAHANCA10 *N* (%)	DAHANCA19 *N* (%)	Dutch cohort *N* (%)	Singapore cohort *N* (%)
Number of patients	1183 (100)	617 (52)	81 (7)	485 (41)	597 (100)	235 (100)
Age in years						
Median (range)	60 (27–90)	61 (27–90)	56 (35–80)	59 (32–80)	63 (20–89)	51 (21–91)
Sex						
Male	940 (79)	481 (78)	63 (78)	396 (82)	399 (67)	182 (77)
Female	243 (21)	136 (22)	18 (22)	89 (18)	198 (33)	53 (23)
Dose						
66 Gy	765 (65)	466 (76)	56 (69)	243 (50)	496 (83)	0 (0)
68 Gy	383 (32)	148 (24)	25 (31)	210 (43)	35 (6)	0 (0)
70 Gy	35 (3)	3 (0)	0 (0)	32 (7)	66 (11)	235 (100)
Volume surrogate						
T1a N0 M0 glottic larynx	159 (13)	159 (26)	0 (0)	0 (0)	78 (13)	13 (1)
Tx N0 M0	215 (18)	145 (23)	19 (23)	51 (11)	221 (37)	11 (18)
Tx N1–3M0	809 (68)	13 (51)	62 (77)	434 (89)	298 (50)	211 (22)
Chemotherapy						
No	714 (60)	505 (82)	81 (100)	128 (26)	445 (75)	53 (23)
Yes	469 (40)	112 (18)	0 (0)	357 (74)	152 (25)	182 (78)
*Endpoint frequency*						
Acute dysphagia						
Moderate/severe	730 (62)	304 (49)	53 (65)	373 (77)	—	—
Severe	410 (35)	153 (25)	29 (36)	228 (47)	—	—
Mucositis						
Moderate/severe	870 (74)	395 (64)	63 (78)	412 (85)	170 (28)	49 (21)
Severe	41 (3)	19 (3)	3 (4)	19 (4)	—	—
Late dysphagia						
Moderate/severe	143 (12)	54 (9)	14 (17)	75 (15)	—	—
Severe	60 (5)	23 (4)	4 (5)	33 (7)	—	—
Fibrosis						
Moderate/severe	628 (53)	97 (48)	54 (67)	277 (57)	—	—
Severe	166 (14)	66 (11)	12 (15)	88 (18)	—	—
Fibrosis/atrophy						
Moderate/severe	822 (69)	404 (65)	67 (83)	351 (72)	—	—
Severe	202 (17)	81 (13)	15 (19)	106 (22)	—	—
Xerostomia						
Moderate/severe	755 (64)	368 (60)	67 (83)	320 (66)	—	—
Severe	276 (23)	149 (24)	39 (48)	88 (18)	—	—
Tube feeding at 6 months	78 (7)	41 (7)	8 (10)	29 (6)	—	—

#### GWAS–QQ and Manhattan plots

**Fig. 1:**
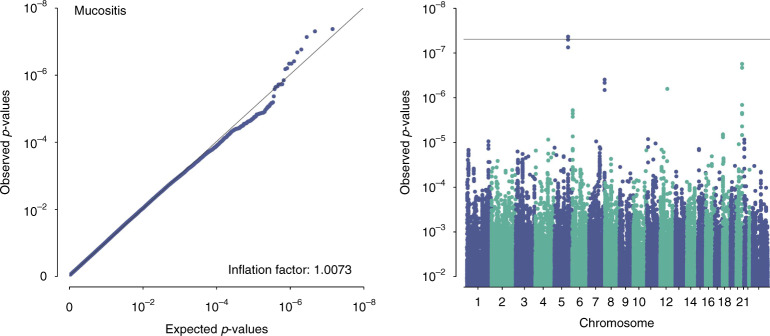
GWAS results for moderate/severe mucositis. QQ plot (left) and Mangattan plot (right).

The 7,178,424 SNPs were tested for associations with acute dysphagia, mucositis, late dysphagia, xerostomia, skin fibrosis, fibrosis/atrophy, tube feeding at 6 months, STAT_acute_, STAT_late_ and STAT_global_. The first 7 endpoints were analysed as binary outcomes, and the first six of these were analysed at two different cut points, ‘moderate-to-severe’ and ‘severe’ (Supplementary Table 1). Due to a low number of severe events, mucositis was only evaluated as ‘moderate-to-severe’. The analysis identified a significant association for mucositis with QQ and Manhattan plots shown in Fig. 1. QQ and Manhattan plots for all endpoints are shown in Supplementary Fig. 3.

In moderate-to-severe acute dysphagia, an imputed single SNP, rs2024705 on chromosome X, reached genome-wide significance. Imputation quality was rather poor (info = 0.44). This SNP represents an intron variant in LINC00632 (long intergenic non-protein-coding RNA 632) and was considered a potential false-positive finding.

#### Mucositis

**Fig. 2:**
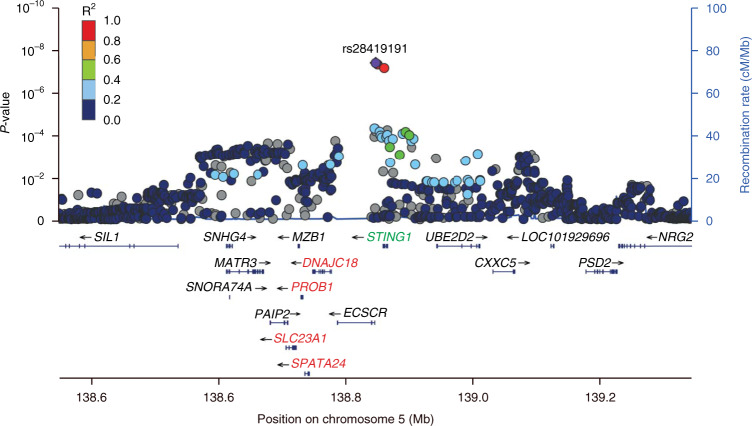
Locus Zoom plot for a locus on chromosome 5 associated with moderate/severe mucositis. Variants in orange or red colour are in Linkage Disequilibrium with the specified SNP rs28419191. The locus is in cis-eQTL with genes marked in red and in sQTL with gene marked in green.

Three SNPs (rs28419191, rs10875554 and rs1131769) tagged a locus on chromosome 5 that reached genome-wide significance for association with radiation-induced mucositis (Table 2 and Fig. 2). The strongest association was found for effect allele C (allele frequency 0.87) of rs28419191 with OR = 2.27; 95% CI 1.70–3.05; *p* = 4.4 ∙ 10^−8^; *p*_Bayes_ = 0.068. The SNPs were well imputed (imputation info score range 0.95–0.96) and in close LD with each other (Supplementary Fig. 4A). The locus spans an area from 4 kb upstream of the ECSCR gene (rs28419191 and rs10875554) to an exon in the *STING1* gene, where rs1131769T > C represents a missense variant changing the codon from H (His) > R (Arg). The locus is in cis-eQTL with *DNAJC18*, *PROB1*, *SLC23A1*, *SPATA24* and in sQTL with *STING1* [30]. *DNAJC18*, *PROB1*, *SLC23A1* and *SPATA24* have a similar expression pattern across tissue types and *ECSCR* and *STING1* share a different expression pattern (Supplementary Fig. 5).

**Table 2:** Summary results of the association between a locus on chromosome 5 and RT-induced mucositis in discovery, replication and meta-analysis.

SNP	Discovery study (*N* = 1183)					Replication study (Dutch) (*N* = 597)				Meta-analysis (*N* = 1780)			
	Effect allele	MA (MAF)^a^	OR^b^	SE^c^	*p*-value	MA (MAF)^a^	OR	SE	*p*-value	Pooled OR	Pooled SE	Pooled *p*-value	I^2^
rs28419191	C	T (0.13)	2.272	0.344	4.4 × 10^−8^	T (0.13)	1.550	0.392	0.035	1.958	0.259	3.6 × 10^−14^	47.8%
rs1131769	C	T (0.13)	2.209	0.329	7.6 × 10^−8^	T (0.12)	1.650	0.349	0.016	1.946	0.239	4.3 × 10^−16^	26.4%
rs10875554	C	T (0.13)	2.248	0.331	5.1 × 10^−8^	Data not available in replication cohort							

^a^*MA (MAF)* Minor allele (frequency).

^b^*OR* odds ratio.

^c^*SE* standard error.

ECSCR (Endothelial Cell Surface expressed Chemotaxis and apoptosis Regulator protein, previously known as Endothelial Cell-Specific Molecule 2 (ECSM2)) is expressed mainly in endothelial cells and blood vessels [31] and is involved in the regulation of chemotaxis and apoptosis through modulation of various pathways including EGFR, VEGF and FGFR signaling [32, 33].

*STING1* (STimulator of INterferon response cGAMP interactor 1, previously known as transmembrane protein 173 (TMEM173)) has several splice variants. It is critically involved in the β-interferon synthesis and inflammation/immune system function [34] and non-canonical activation of a STING signalling pathway has been observed following DNA damage [35].

*DNAJC18* is a member (C18) of the DnaJ heat shock protein family (Hsp40) [36]. The transcripts of this family function as chaperone molecules protecting other cellular proteins across pro- and eukaryotic species [37].

*PROB1* (PROline rich Basic protein 1) is a protein-coding gene [36]. Its function in humans is not known, although one study has identified a possible association between alterations in this gene and the eye disorder keratoconus [38].

*SLC23A1* (SoLute Carrier family 23 member 1, formerly SVCT1) is a member of the solute carrier (SLC) group of proteins and is involved in the transport of ascorbic acid (Vitamin C) across the cell membrane [39, 40].

*SPATA24* (spermatogenesis associated 24) also a protein-coding gene, expressed in several tissues, in particular in the testes and prostate. It is preserved in several mammals where it plays a role in Cell differentiation as well as spermatogenesis [39].

The study did not identify other loci reaching genome-wide significance. A near-significant association was found between a locus on chromosome 6 and STATacute, available in Supplementary Figs. 4B, 6 and in Supplementary Table 3, where a full list of variants reaching *p*-values < 10^−5^ is available for all endpoints.

### Replication study

#### Dutch cohort

##### Genotyping and patient characteristics

Among 1102 HNC samples, 957 patients and 6,334,277 SNPs passed imputation process and quality controls. The Dutch cohort includes HNC patients treated with both definitive and postoperative RT. Phenotypic quality control yielded 597 patients who were treated with definitive RT and met the remaining eligibility criteria matching the discovery study. Table 1 presents the characteristics of the Dutch replication population.

##### Replication and meta-analysis

The discovery study identified three variants significantly and 27 variants suggestively associated with mucositis. Two out of 3 genome-wide significant and 21 out of 27 suggestively significant variants were available for analysis in the replication study. We found the rs28419191*C allele with an OR of 1.55 (95% CI 1.03–2.32; *p* < 0.035) and rs1131769*C allele with an OR of 1.65 (95% CI 1.10–2.47; *p* < 0.015) significantly associated with increased risk of mucositis in the replication cohort (Table 2). None of the 21 suggestive SNPs from the discovery study was associated with mucositis in the replication cohort (Supplementary Table 3, sheet “Mucositis”). Next, we performed a meta-analysis on summary data for the discovery and replication studies, the combined subjects amounting to 1780 patients. The pooled OR for rs28419191*C allele was 1.96 (95% CI 1.45–2.46; *p*_meta-analysis_ = 3.67 × 10^−14^) and that for rs1131769*C allele was 1.95 (95% CI 1.48–2.41; *p*_meta-analysis_ = 4.34 × 10^−16^) and thus significantly associated to mucositis (Table 2).

##### Singapore cohort

In 235 samples, 49 patients with nasopharyngeal carcinoma experienced moderate-severe mucositis. Patient characteristics are presented in Table 1. In the locus eligible for analysis, rs28419191 and rs10875554 were unavailable for analysis and only rs1131769, was therefore analysed. This variant failed to replicate with an OR = 0.94 (95% CI 0.46–1.92; *p* = 0.86). The genotyping quality was good (0.99). However, the effect allele C had a lower frequency than in the DAHANCA and Dutch cohorts with 0.77 vs 0.87 and 0.87, respectively.

## Discussion

Many studies in radiogenomics have been conducted since the turn of the millennium and several genomic loci have been identified as significantly associated with RT-induced toxicity. Up to now, breast, prostate, gynaecological and lung cancer cohorts are represented in hypothesis-driven studies of associations between SNPs and RT-induced toxicity. In non-hypothesis-driven GWA studies, breast and prostate cancer cohorts have been explored [8–11] and two fairly recent studies explored loci associated with temporal lobe injury [12] and oral mucositis [13] reaching *p*-values of 10^−7^ after RT for nasopharyngeal carcinoma.

The present study is the first GWAS study to explore associations with RT-induced toxicity in non-nasopharyngeal HNC cohorts. Previous studies in breast cancer cohorts have indicated that genetic variations may predispose to both an endpoint-specific as well as a more general susceptibility of developing RT-induced toxicity [41, 42] and hence supports the idea that bringing a cancer site with new RT-induced toxicity data to the research field of radiogenomics may contribute both site-specific and general value. The study identified and replicated a locus on chromosome 5 in the *STING1* gene to be significantly associated with RT-induced mucositis and meta-analysis confirmed this association. The expression pattern falls into two groups, rendering it likely that this locus could be involved by either splice variants in the STING1 pathway with which the locus is in sQTL or the group of genes (*DNAJC18*, *PROB1*, *SLC23A1* and *SPATA24)* with which the locus is in cis-eQTL (Supplementary Fig. 5).

An effort was done to replicate this finding in an Asian ancestry cohort from Singapore using whole-exome sequencing data. However, it failed to replicate due to most likely a different tumour site (nasopharynx) bringing a different distribution of RT-induced toxicity, a low sample size and the fact that only one variant, rs1131769, was available for analysis and this had a lower minor allele frequency than in the European ancestry cohorts studied here. In accordance with this chain of reasoning, the loci reaching low *p*-values in the present study were different from the ones identified in [13] exploring RT-induced oral mucositis in patients with both a different tumour site and ancestry (nasopharyngeal cancers in Chinese patients).

We did not identify SNPs significantly associated with any other acute and late endpoints. This may own to the sample size of 1183 patients in the discovery study, which was a limitation to the study as it rendered the identification of low-penetrance genetic variants less likely.

The significance level in the discovery phase was set to *p* < 5 × 10^−8^ and thus was not adjusted for testing multiple endpoints. This was a pragmatic choice that follows consensus in GWA studies and further was based on the considerations that the replication phase included genetic variants suggestively significant, and as the multiple endpoints tested are not individually independent, a Bonferroni estimate of a corrected *p*-value may increase the risk of false-negative discoveries to a larger extent than it will decrease the risk of false-positive findings. However, we do acknowledge this limitation and recommend that the finding in our study should be validated in other independent comparable cohorts before considered conclusive.

It is our hope that this study can serve such future independent studies as summary data for associations reaching *p*-values < 10^−5^ are published in Supplementary Table 3 and may serve as a reference for directionality, effect size and endpoint association. By design, the present study analysed the association between individual SNPs and endpoints. The complexity of various types of epistasis and other causes for modification of gene function (epigenetics, gene-environment interactions) were beyond the scope.

One of the more robust findings in radiogenomics is an association between *ATM* variants and RT-induced morbidity. A meta-analysis across breast and prostate cancer cohorts identified rs1801516 *A associated with ORs of 1.5 or 1.2 for being in the upper quartile of acute and late toxicity, respectively [9]. We did not identify associations between *ATM* variants and endpoints in this study which may own to the limitation of sample size, not rendering identification of variants in this range of effect size likely.

In conclusion, this is the first study to identify and replicate a locus associated with RT-induced mucositis in HNC patients with a laryngeal or oro/hypopharyngeal tumour site. It serves to extend the field of radiogenomics and adds a piece to the increasing evidence that an individual genetic predisposition is a contributory factor in the development of toxicity after RT. Collaborative efforts of combining cohorts of HNC patients with solid data for RT-induced morbidity would enable identification of genetic variants with lower penetrance, possibilities for validation and in the longer run, to add biology variables to Normal Tissue Complication Models. Such efforts are warranted under the auspices of the Head and Neck Cancer Group in the Radiogenomics Consortium.

### Reporting guidelines

The study was reported complying with the STROGAR guidelines for radiogenomic studies.

### Web resources

dbSNP, https://www.ncbi.nlm.nih.gov/SNP/, GAS power calculator, http://csg.sph.umich.edu/abecasis/cats/gas_power_calculator/index.html, GATK, https://gatk.broadinstitute.org/hc/en-us, The Genotype-Tissue Expression (GTEx) Project, gtexportal.org, IMPUTE2, http://mathgen.stats.ox.ac.uk/impute/impute_v2.html, LocusZoom, http://locuszoom.org/, National Institute of Health LD analysis and plot, https://analysistools.nci.nih.gov/LDlink/, PLINK, https://www.cog-genomics.org/plink2, R Software, https://www.r-project.org/, SHAPEIT, https://mathgen.stats.ox.ac.uk/genetics_software/shapeit/shapeit.html, SNPedia, https://www.snpedia.com/index.php/SNPedia, SNPTEST, https://mathgen.stats.ox.ac.uk/genetics_software/snptest/snptest.html.

### Reporting summary

Further information on research design is available in the Nature Research Reporting Summary linked to this article.

## Supplementary information

Supplementary Material

Reporting Summary

Supplementary Table 3

## Data Availability

Summary data for associations reaching *p*-values < 10^−5^ are available in Supplementary Table 3.

## References

[1] Blanchard P, Baujat B, Holostenco V, Bourredjem A, Baey C, Bourhis J (2011). Meta-analysis of chemotherapy in head and neck cancer (MACH-NC): a comprehensive analysis by tumour site. Radiother Oncol.

[2] Bourhis J, Overgaard J, Audry H, Ang KK, Saunders M, Bernier J (2006). Hyperfractionated or accelerated radiotherapy in head and neck cancer: a meta-analysis. Lancet.

[3] Overgaard J, Jovanovic A, Godballe C, Grau Eriksen J (2016). The Danish Head and Neck Cancer database. Clin Epidemiol.

[4] Brodin NP, Kabarriti R, Garg MK, Guha C, Tomé WA (2018). Systematic review of normal tissue complication models relevant to standard fractionation radiation therapy of the Head and Neck Region Published After the QUANTEC Reports. Int J Radiat Oncol Biol Phys.

[5] Rosenstein BS, West CM, Bentzen SM, Alsner J, Andreassen CN, Azria D (2014). Radiogenomics: radiobiology enters the era of big data and team science. Int J Radiat Oncol Biol Phys.

[6] West C, Rosenstein BS, Alsner J, Azria D, Barnett G, Begg A (2010). Establishment of a radiogenomics consortium. Int J Radiat Oncol Biol Phys.

[7] West C, Rosenstein BS (2010). Establishment of a radiogenomics consortium. Radiother Oncol.

[8] Herskind C, Talbot CJ, Kerns SL, Veldwijk MR, Rosenstein BS, West CML (2016). Radiogenomics: a systems biology approach to understanding genetic risk factors for radiotherapy toxicity?. Cancer Lett.

[9] Andreassen CN, Rosenstein BS, Kerns SL, Ostrer H, De Ruysscher D, Cesaretti JA (2016). Individual patient data meta-analysis shows a significant association between the ATM rs1801516 SNP and toxicity after radiotherapy in 5456 breast and prostate cancer patients. Radiother Oncol.

[10] Kerns SL, Fachal L, Dorling L, Barnett GC, Baran A, Peterson DR (2019). Radiogenomics consortium genome-wide association study meta-analysis of late toxicity after prostate cancer radiotherapy. J Natl Cancer Inst..

[11] Lee S, Kerns S, Ostrer H, Rosenstein B, Deasy JO, Oh JH (2018). Machine learning on a genome-wide association study to predict late genitourinary toxicity after prostate radiation therapy. Int J Radiat Oncol Biol Phys.

[12] Wang T-M, Shen G-P, Chen M-Y, Zhang J-B, Sun Y, He J (2019). Genome-wide association study of susceptibility loci for radiation-induced brain injury. J Natl Cancer Inst.

[13] Yang D-W, Wang T-M, Zhang J-B, Li X-Z, He Y-Q, Xiao R (2020). Genome-wide association study identifies genetic susceptibility loci and pathways of radiation-induced acute oral mucositis. J Transl Med.

[14] Sumner W, Ray X, Sutton L, Rebibo D, Marincola F, Sanghvi P (2021). Gene alterations as predictors of radiation-induced toxicity in head and neck squamous cell carcinoma. J Transl Med.

[15] Overgaard J, Hoff CM, Hansen HS, Specht L, Overgaard M, Lassen P (2018). DAHANCA 10—Effect of darbepoetin alfa and radiotherapy in the treatment of squamous cell carcinoma of the head and neck. A multicenter, open-label, randomized, phase 3 trial by the Danish head and neck cancer group. Radiother Oncol.

[16] Eriksen JG, Maare C, Johansen J, Primdahl H, Evensen J, Kristensen CA (2015). OC-009: Update of the randomised phase III trial DAHANCA 19: -primary C-RT or RT and zalutumumab for squamous cell carcinomas of head and neck. Radiother Oncol.

[17] The Danish Head and Neck Cancer Group. 2021. https://www.dahanca.dk/.

[18] LENT SOMA tables. Radiother Oncol. 1995;35:17–60. 7569012

[19] Barnett GC, West CML, Coles CE, Pharoah PDP, Talbot CJ, Elliott RM (2012). Standardized Total Average Toxicity score: a scale- and grade-independent measure of late radiotherapy toxicity to facilitate pooling of data from different studies. Int J Radiat Oncol Biol Phys.

[20] Amos CI, Dennis J, Wang Z, Byun J, Schumacher FR, Gayther SA (2017). The OncoArray Consortium: A network for understanding the genetic architecture of common cancers. Cancer Epidemiol Biomark Prev.

[21] Wakefield J (2007). A Bayesian measure of the probability of false discovery in genetic epidemiology studies. Am J Hum Genet.

[22] Phelan CM, Kuchenbaecker KB, Tyrer JP, Kar SP, Lawrenson K, Winham SJ (2017). Identification of 12 new susceptibility loci for different histotypes of epithelial ovarian cancer. Nat Genet.

[23] R Core Team. R: A language and environment for statistical computing. Vienna: R Foundation for Statistical Computing. 2021. https://www.r-project.org/.

[24] Marchini J, Howie B, Myers S, McVean G, Donnelly P (2007). A new multipoint method for genome-wide association studies by imputation of genotypes. Nat Genet.

[25] Kerns SL, de Ruysscher D, Andreassen CN, Azria D, Barnett GC, Chang-Claude J (2014). STROGAR—STrengthening the Reporting Of Genetic Association studies in Radiogenomics. Radiother Oncol.

[26] Skol AD, Scott LJ, Abecasis GR, Boehnke M (2006). Joint analysis is more efficient than replication-based analysis for two-stage genome-wide association studies. Nat Genet.

[27] Price AL, Patterson NJ, Plenge RM, Weinblatt ME, Shadick NA, Reich D (2006). Principal components analysis corrects for stratification in genome-wide association studies. Nat Genet.

[28] Willer CJ, Li Y, Abecasis GR (2010). METAL: fast and efficient meta-analysis of genomewide association scans. Bioinformatics.

[29] Poplin R, Ruano-Rubio V, DePristo M, Fennell T, Carneiro M, Van der Auwera G, et al. Scaling accurate genetic variant discovery to tens of thousands of samples. BioRxiv. 2017. 10.1101/201178.

[30] Aguet F, Barbeira AN, Bonazzola R, Brown A, Castel SE, Jo B (2020). The GTEx Consortium atlas of genetic regulatory effects across human tissues. Science.

[31] Ma F, Zhang D, Yang H, Sun H, Wu W, Gan Y (2009). Endothelial cell-specific molecule 2 (ECSM2) modulates actin remodeling and epidermal growth factor receptor signaling. Genes Cells.

[32] Verma A, Bhattacharya R, Remadevi I, Li K, Pramanik K, Samant GV (2010). Endothelial cell-specific chemotaxis receptor (ecscr) promotes angioblast migration during vasculogenesis and enhances VEGF receptor sensitivity. Blood.

[33] Shi C, Lu J, Wu W, Ma F, Georges J, Huang H (2011). Endothelial cell-specific molecule 2 (ECSM2) localizes to cell-cell junctions and modulates bFGF-directed cell migration via the ERK-FAK pathway. PLoS ONE.

[34] Nandakumar R, Windross SJ, Paludan SR. Intercellular communication in the innate immune system through the cGAS-STING pathway. Methods Enzymol. 2019;625:1–11. 10.1016/bs.mie.2019.07.007 31455521

[35] Dunphy G, Flannery SM, Almine JF, Connolly DJ, Paulus C, Jønsson KL (2018). Non-canonical activation of the DNA sensing adaptor STING by ATM and IFI16 mediates NF-κB signaling after nuclear DNA damage. Mol Cell.

[36] Howe KL, Achuthan P, Allen J, Allen J, Alvarez-Jarreta J, Ridwan Amode M (2021). Ensembl 2021. Nucleic Acids Res.

[37] Qiu XB, Shao YM, Miao S, Wang L (2006). The diversity of the DnaJ/Hsp40 family, the crucial partners for Hsp70 chaperones. Cell Mol Life Sci.

[38] Karolak JA, Gambin T, Pitarque JA, Molinari A, Jhangiani S, Stankiewicz P (2017). Variants in SKP1, PROB1, and IL17B genes at keratoconus 5q31.1-q35.3 susceptibility locus identified by whole-exome sequencing. Eur J Hum Genet.

[39] Tweedie S, Braschi B, Gray K, Jones TEM, Seal RL, Yates B (2021). Genenames.org: The HGNC and VGNC resources in 2021. Nucleic Acids Res.

[40] Tsukaguchi H, Tokui T, Mackenzle B, Berger UV, Chen XZ, Wang Y (1999). A family of mammalian Na+-dependent L-ascorbic acid transporters. Nature.

[41] Bentzen SM, Overgaard M, Overgaard J (1993). Clinical correlations between late normal tissue endpoints after radiotherapy: Implications for predictive assays of radiosensitivity. Eur J Cancer.

[42] Andreassen CN, Alsner J, Overgaard J, Herskind C, Haviland J, Owen R (2005). TGFB1 polymorphisms are associated with risk of late normal tissue complications in the breast after radiotherapy for early breast cancer. Radiother Oncol.

