# Supplementary material for: A genome-wide association study of radiotherapy induced toxicity in head and neck cancer patients identifies a susceptibility locus associated with mucositis
Source: Br J Cancer. 2022 Jan 17;126(7):1082–90. doi: 10.1038/s41416-021-01670-w (PMC8980077; doi:10.1038/s41416-021-01670-w)

Supplementary Material

A Genome-Wide Association Study of Radiotherapy Induced Toxicity in Head and Neck Cancer Patients Identifies a Susceptibility Locus Associated with Mucositis

Line M H Schack, Elnaz Naderi, Laura Fachal, Leila Dorling, Craig Luccarini, Alison M Dunning, The Head and Neck Group of the Radiogenomics Consortium, The Danish Head and Neck Cancer Group (DAHANCA), Enya H W Ong, Melvin L K Chua, Johannes A Langendijk, Behrooz Z Alizadeh, Jens Overgaard, Jesper Grau Eriksen, Christian Nicolaj Andreassen, Jan Alsner

Supplementary Document
Genotyping and imputation quality control 1

Supplementary Table 1
Endpoints and covariates 2

Supplementary Table 2
Flowchart of excluded samples and SNPs for each step of the genotyping- and imputation quality control process 3

Supplementary Table 3
Full list of SNPs performing with p <10^{-5} ..... Separate Excel file

Supplementary Figure 1
Correlations between covariates and endpoints 4

Supplementary Figure 2
Power analysis 14

Supplementary Figure 3
QQ and Manhattan plots 15

Supplementary Figure 4
LD plots 17

Supplementary Figure 5
Gene expression in various tissue types for genes in locus associated with radiation-induced mucositis 18

Supplementary Figure 6
Locus Zoom plot for locus on chromosome 6 19

Supplementary Document

Genotyping and imputation quality control (QC)

Whole-genome DNA was extracted from buffy coats, quantified by Nanodrop and normalized to 100 ng/µl. Genotyping was carried out using the Illumina Infinium OncoArray 500K BeadChip. Wet lab procedures followed Illumina protocols, available at https://support.illumina.com/array/protocols.html. Initial genotype calling was done with GenomeStudio software from Illumina. ≈5000 SNPs on the array are known to not call well and were removed. Based on experience from 56,284 samples with high call rates run, the OncoArray has developed a clustering tool to refine genotype calls from Genome Studio. This and the following QC was done in R. The flowchart of failed samples and SNPs removed can be seen in Supplementary Table 2.

Call rates: As an initial rough selection, samples and SNPs with call rates <80% were excluded. This procedure was then repeated for samples and SNPs with call rates <95%. SNPs with MAF <0.01 were excluded. These SNPs were imputed but not included in analyses if call rate was >0.98.

Heterozygosity: Samples with heterozygosity that fell outside 99.99999 % CI (0.2375123 - 0.2643338, $p=10^{-6}$) were excluded.

Ancestry: Based on a predefined well performing set of ancestry informative markers identified by the OncoArray consortium, ancestry was estimated based on principal components. These were estimated using 23,185 SNPs identified by the OncoArray consortium known to predict ancestry. By means of multidimensional scaling, samples with a European proportion <80% were removed from the dataset.

Gender errors were checked by using a set of 51 well performing chromosome Y SNPs and 10,361 chromosome X SNPs. Number of chromosome Y SNPs and chromosome X heterozygosity was compared and checked for concordance with clinical data for gender.

A p-value of 10^{-7} was set as a cut-off for Hardy-Weinberg equilibrium. Autosomal and chromosome X SNPs were checked separately.

Duplicates and relatives were checked with a set of 7,822 autosomal SNPs known to perform well. A concordance >0.98 as estimated by the identity by state function indicated duplicates or identical twins. Concordance between 0.80-0.98 indicated cryptic relatedness. In case of concordance > 0.80, the sample with the highest number of well genotyped SNPs was kept in the dataset.

Imputed data QC: SNPs with MAF<0.05, SNPs with imputation quality “info” < 0.3 and SNPs that did not pass HWE (p=10-7) were removed.

Supplementary Table 1. Endpoints and covariates.

	Endpoints	Grading	Binary cut point 'moderate/ severe'	Binary cut point 'severe'	STAT scores	
**Acute**	Acute dysphagia	0: no<br>1: light, normal food<br>2: moderate, soft diet<br>3: considerable, only liquid food<br>4: severe, difficult even with liquid food	0-2 vs 3-4	0-3 vs 4	STATacute	STATglobal
	Mucositis	0: no<br>1: erythema<br>2: patchy<br>3: confluent<br>4: ulceration	0-2 vs 3-4	0-3 vs 4		
**Late**	Late dysphagia	0: no<br>1: light, normal food<br>2: moderate, soft diet<br>3: considerable, only liquid food<br>4: severe, difficult even with liquid food	0-2 vs 3-4	0-3 vs 4	STATlate	
	Xerostomia	0: no<br>1: light<br>2: moderate<br>3: severe	0-1 vs 2-3	0-2 vs 3		
	Fibrosis	0: no<br>1: just palpable<br>2: definite increased firmness<br>3: pronounced increased firmness	0-1 vs 2-3	0-2 vs 3		
	Fibrosis / atrophy	Highest score of fibrosis or atrophy<br>Atrophy grading:<br>0: no<br>1: light<br>2: moderate<br>3: severe	0-1 vs 2-3	0-2 vs 3		
**Composite**	Tubefeeding at 6 months	No<br>Yes	No vs Yes			

Covariates	Groups
Sex	Male<br>Female
Dose (Gy)	66<br>68<br>70
Chemotherapy	No<br>Yes
Protocol	Outside protocol<br>DAHANCA 10<br>DAHANCA 19
Volume surrogate	Glottic laryngeal T1N0M0<br>All other TxN0 sites<br>TxN1-3 carcinomas

Supplementary Table 2. Flowchart of excluded samples and genetic variants for each step of the genotyping- and imputation quality control process in the DAHANCA discovery study.

QC step of genotyped data	Samples in dataset	Variants in dataset	Samples removed	Variants removed
Initial data set	1,237	533,631		
Samples with initial call rate <80%	1,225	533,631	12	
Variants with initial call rate <80%		526,692		6,939
Samples with initial call rate <95%	1,221		4	
Variants with initial call rate <95%		522,160		4,532
Variants with maf <0.01 and call rate <0.98%		520,527		1,633
Heterozygosity for autosomal SNPs, $p<10^{-6}$	1,213		8	
Ancestry analysis	1,208		5	
Gender errors	1,200		8	
Hardy-Weinberg equilibrium, $p<10^{-7}$		518,406		2,222 autosomal*<br>48 chromosome X
Remove duplicate controls and relatives	1,183		17	
Final genotype file	1,183	518,406		
Final file for imputation, remove variants in wrong position for imputation	1,183	483,498		34,908
Imputation reference panel 1000 Genomes project final phase, GRCh37	1,183	$14 \cdot 10^{6}$		$6.9 \cdot 10^{6}$
Hardy-Weinberg equilibrium, $p<10^{-7}$	1,183	7,178,430		6
Imputed data set	1,183	7,178,424		

*HWE: Only 2,121 variants were removed due to overlap with variants removed in previous steps

Supplementary Figure 1. Correlations between covariates (sex, total dose, chemotherapy, protocol, volume surrogate) and endpoints in the DAHANCA discovery study.

Moderate/severe acute dysphagia

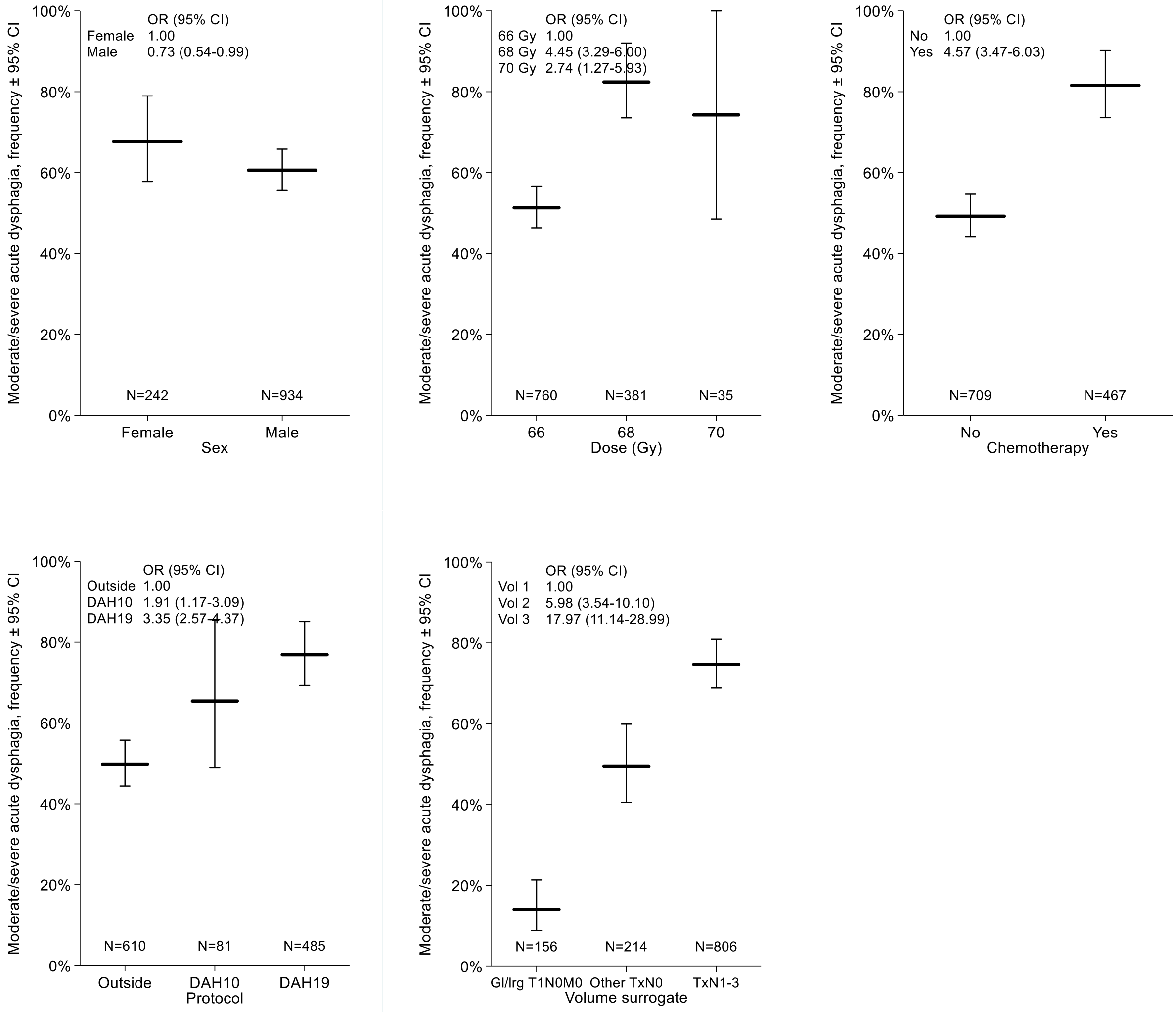

Severe acute dysphagia

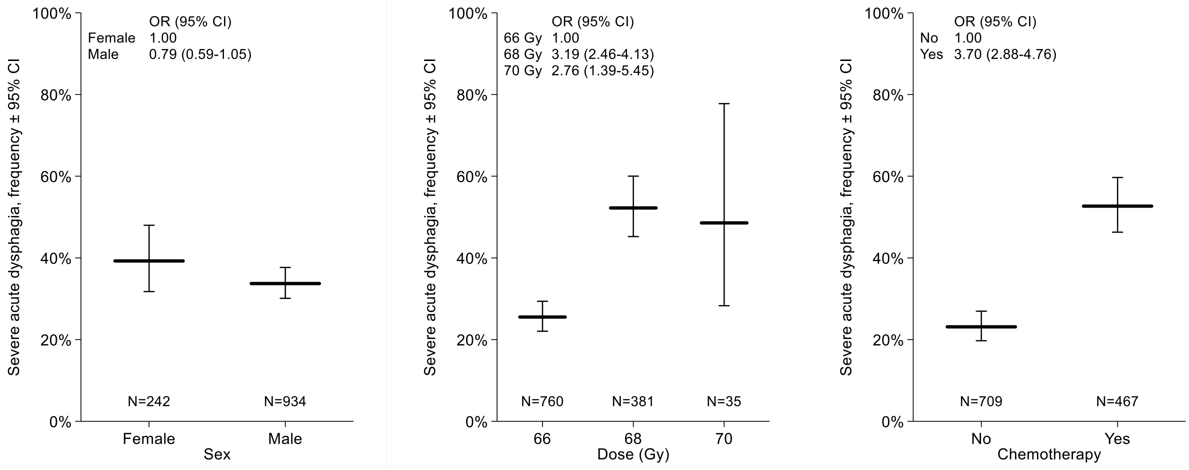

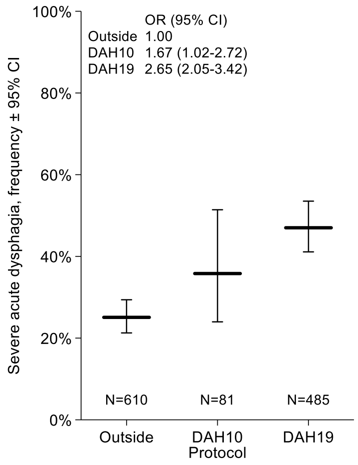

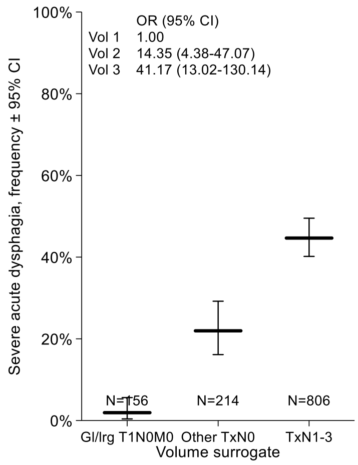

Moderate/severe mucositis

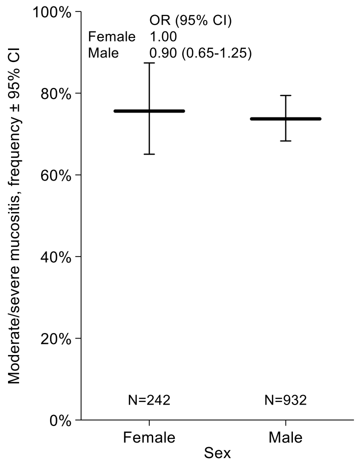

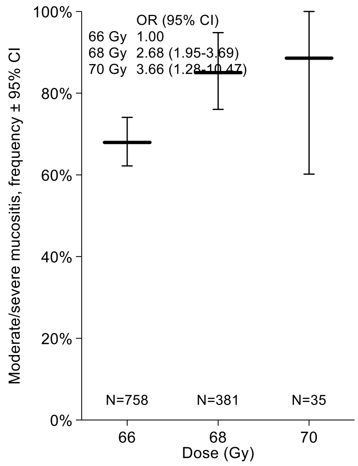

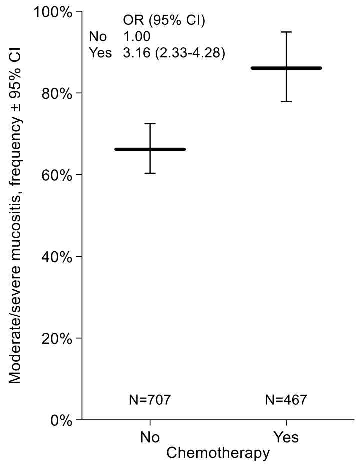

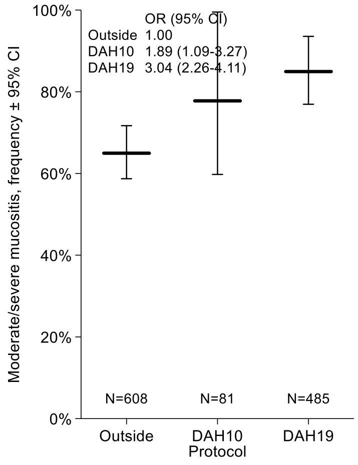

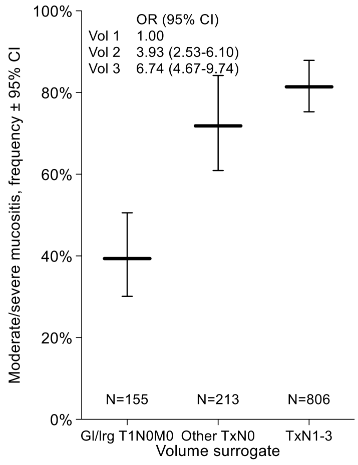

Moderate/severe late dysphagia

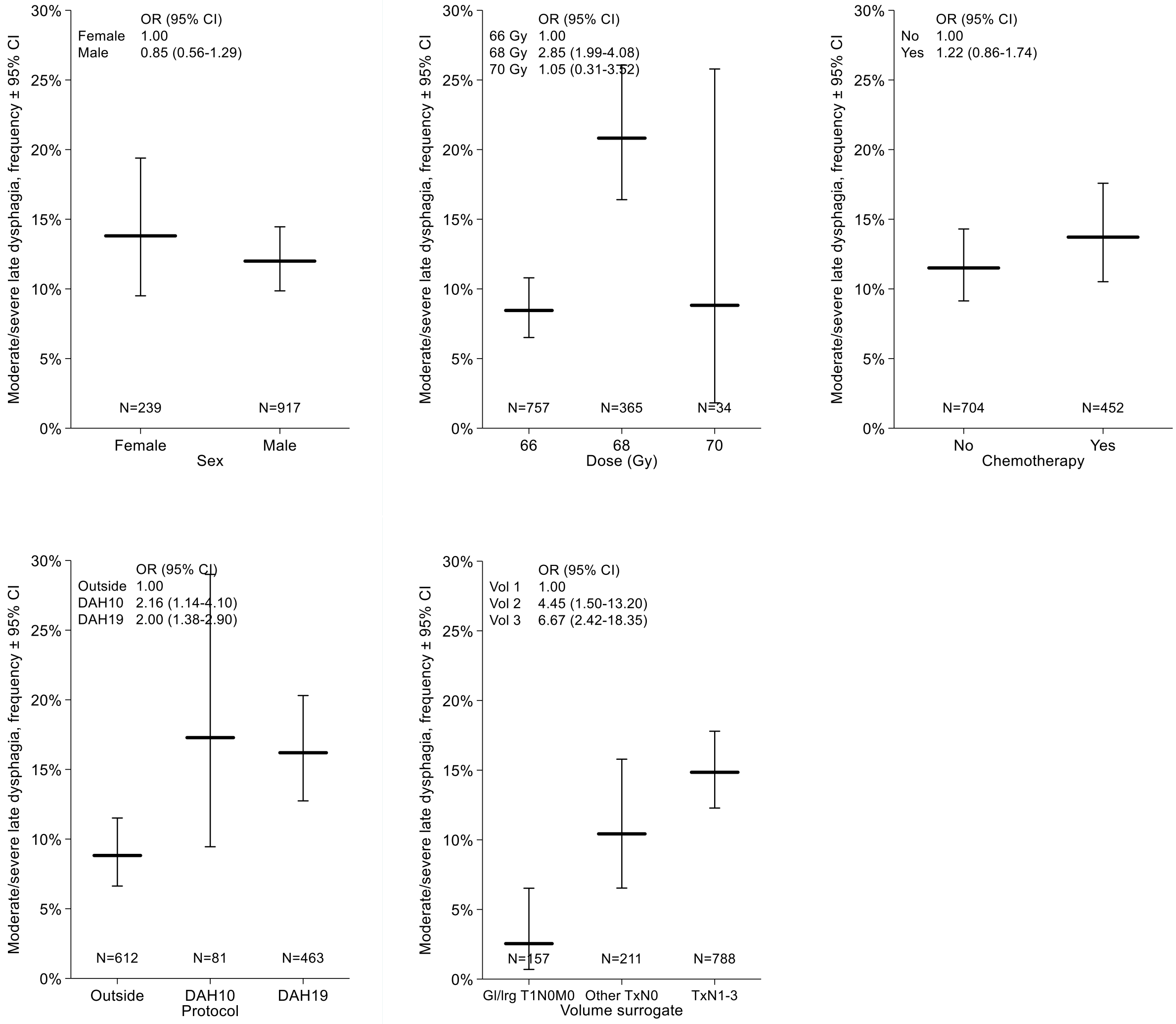

Severe late dysphagia

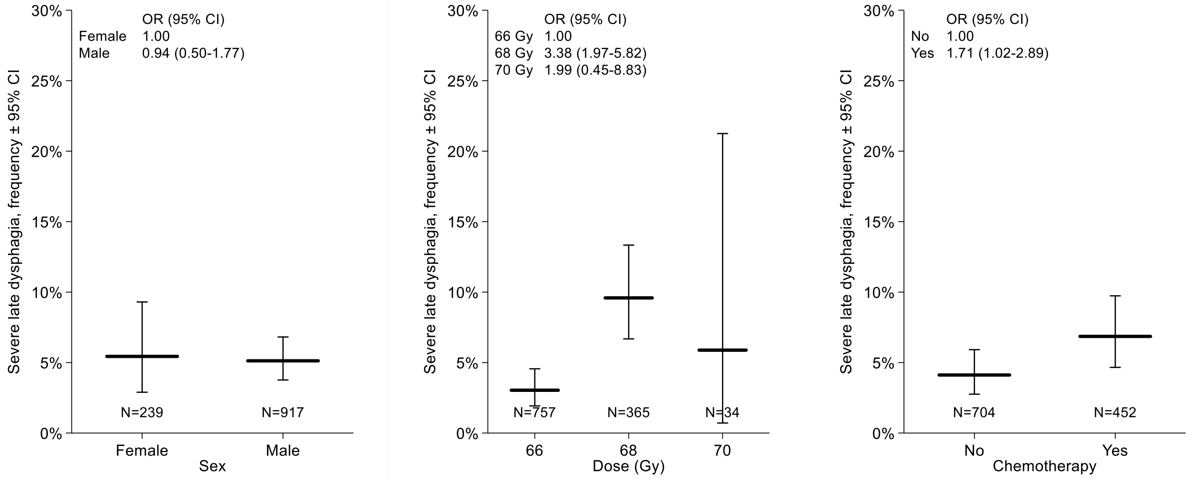

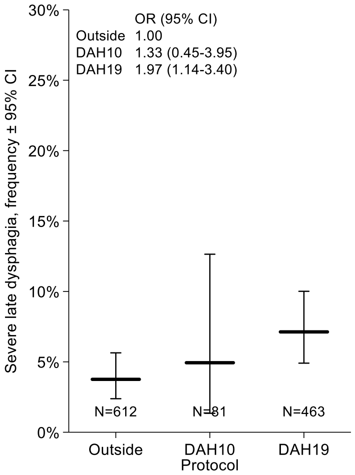

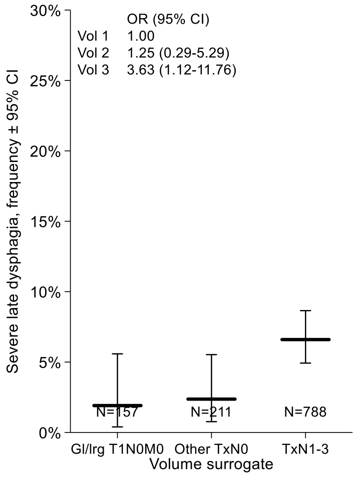

Moderate/severe xerostomia

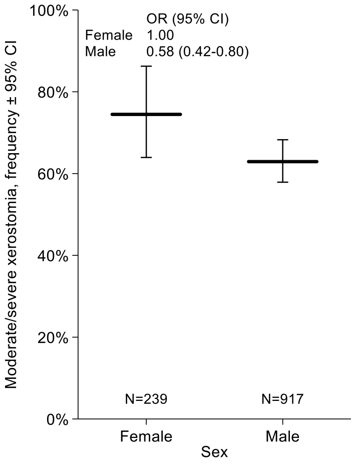

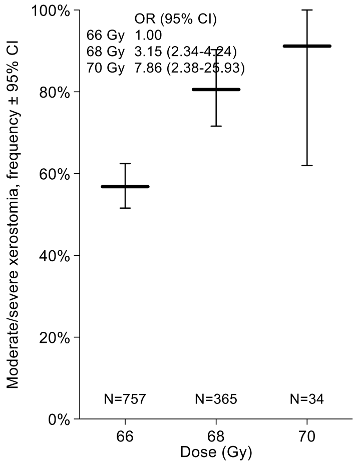

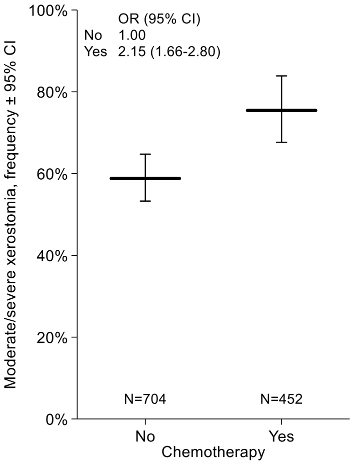

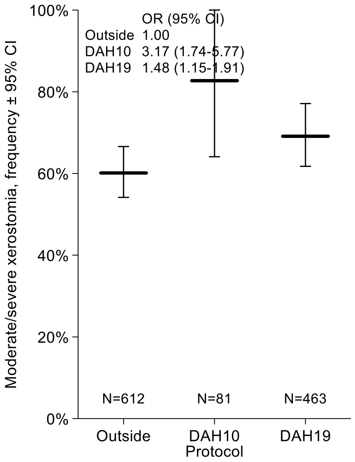

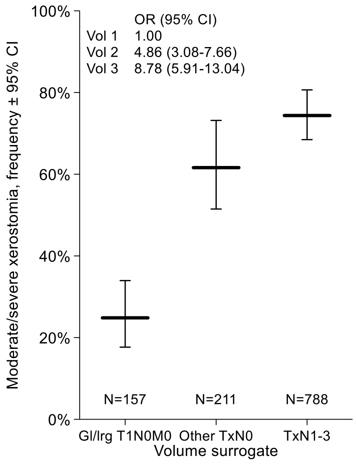

Severe xerostomia

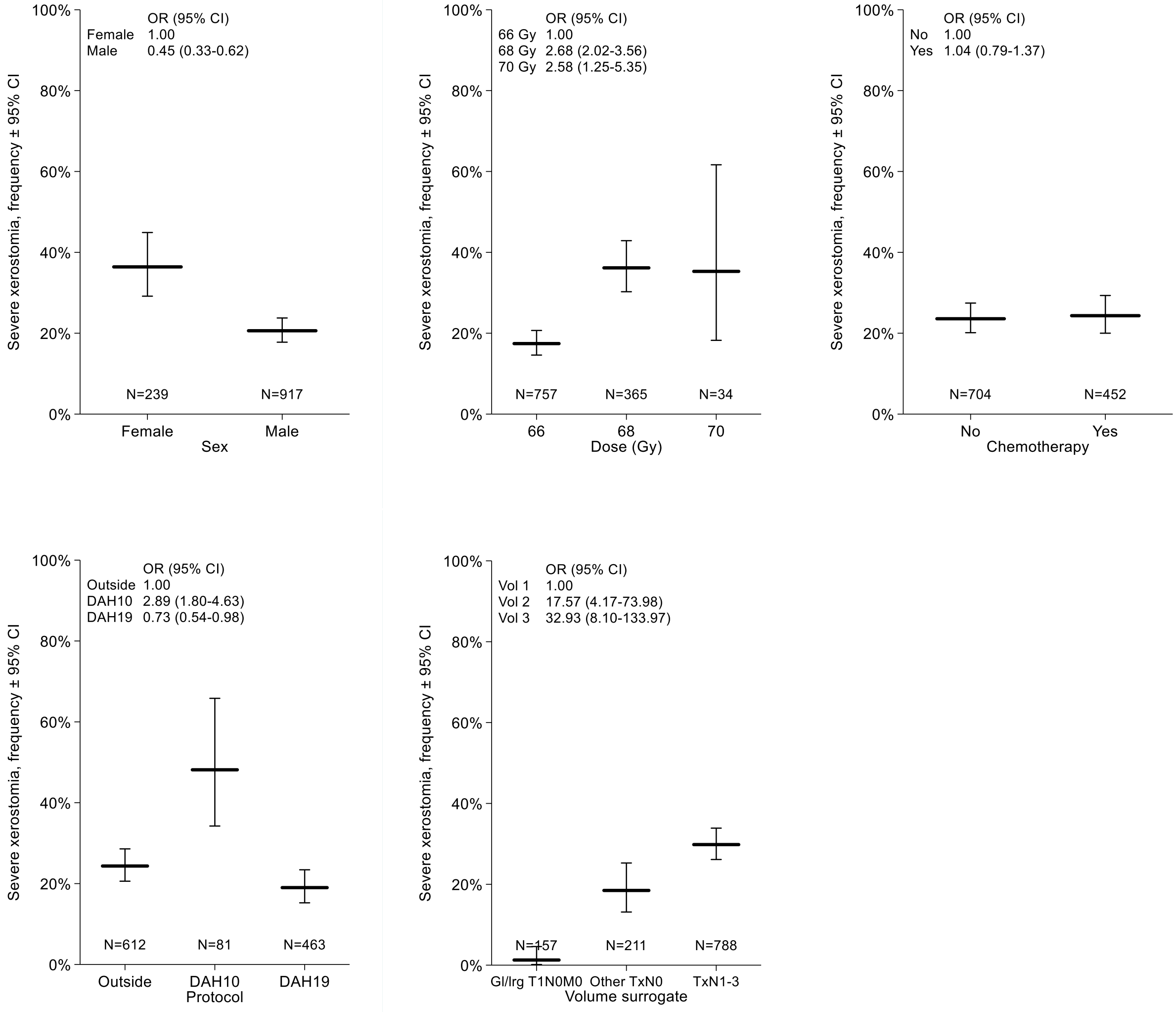

Moderate/severe fibrosis

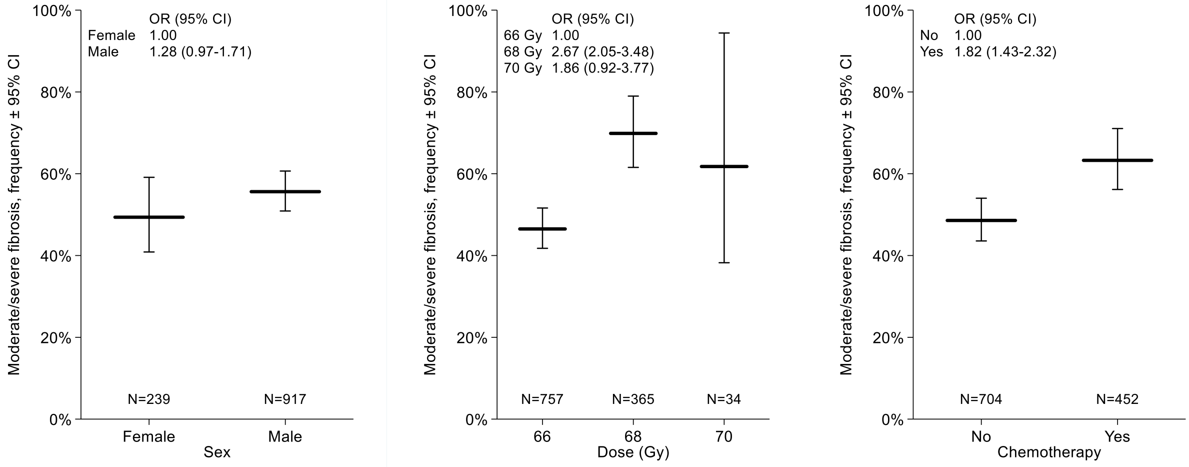

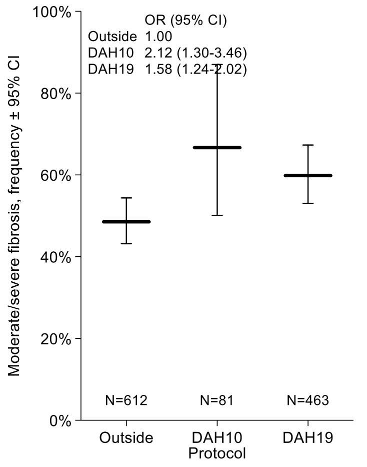

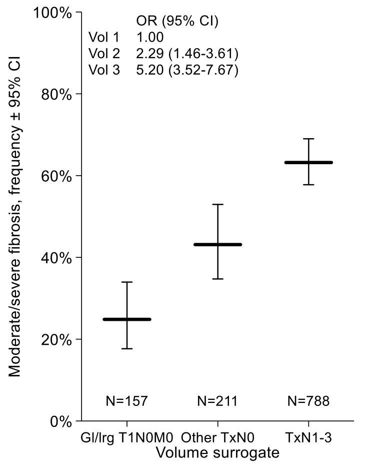

Severe fibrosis

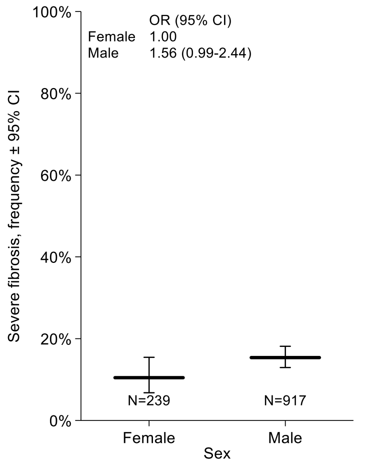

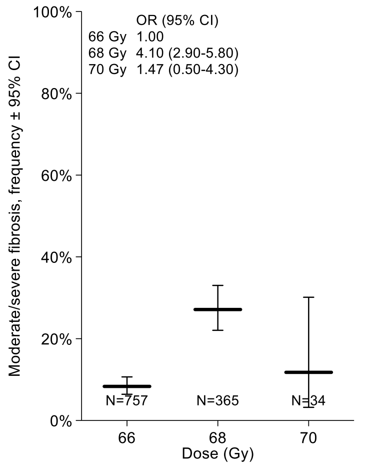

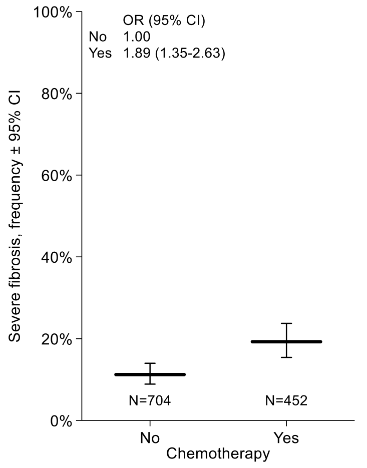

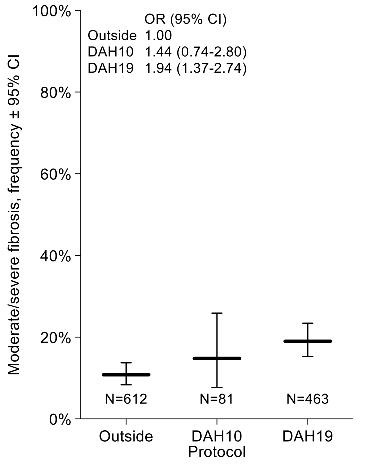

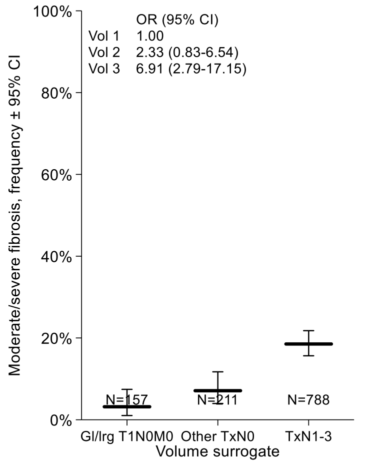

Moderate/severe fibrosis/atrophy

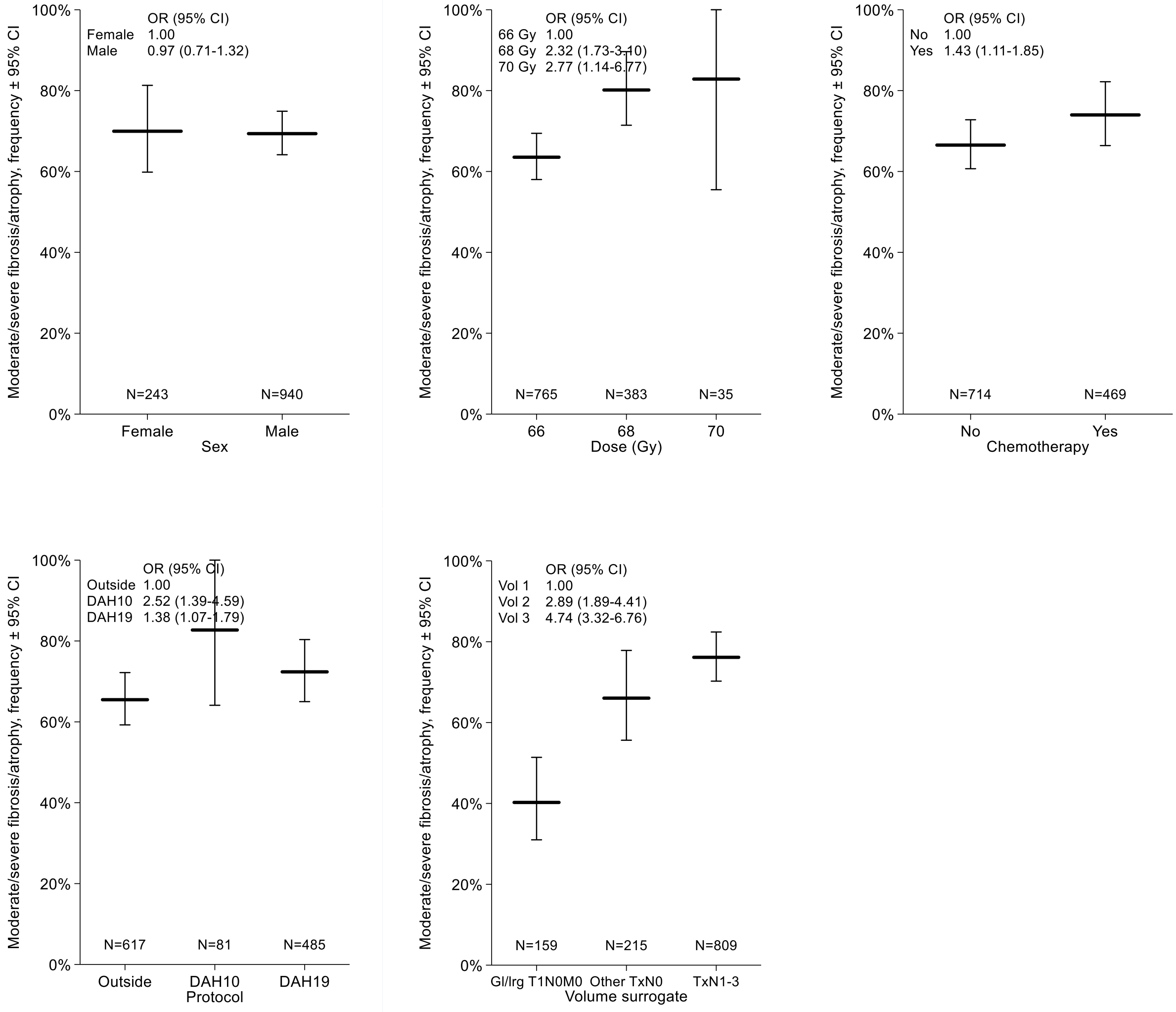

Severe fibrosis/atrophy

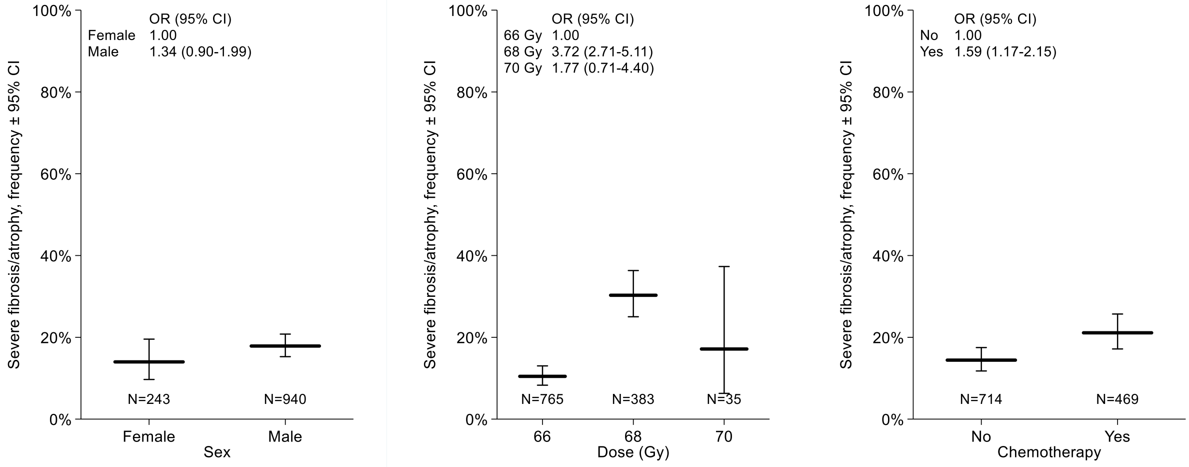

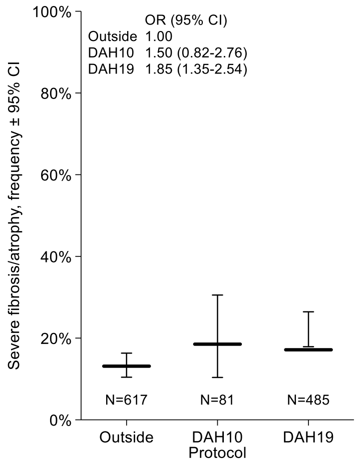

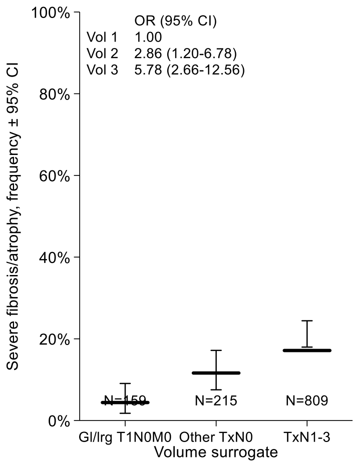

Tubefeeding at 6 months

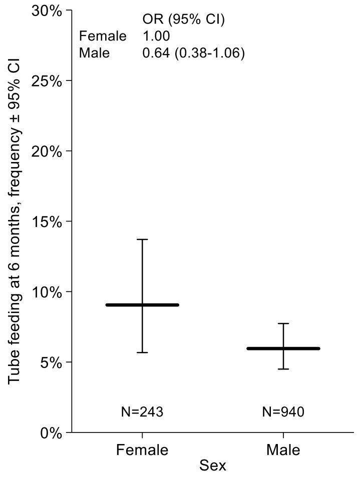

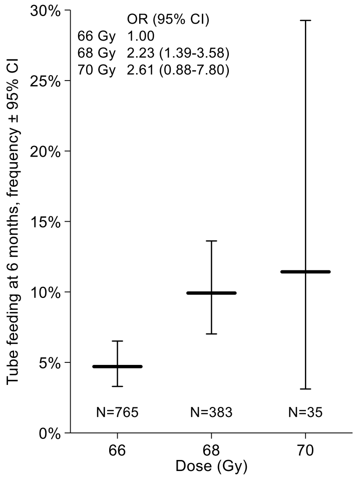

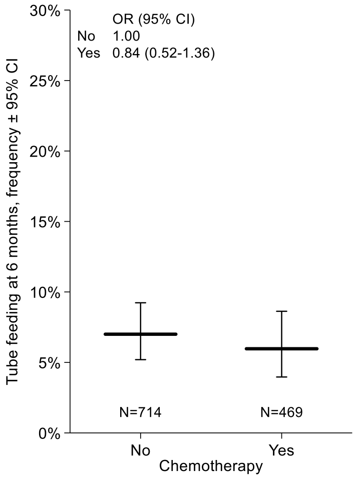

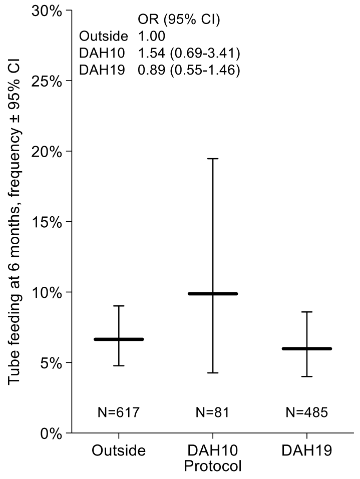

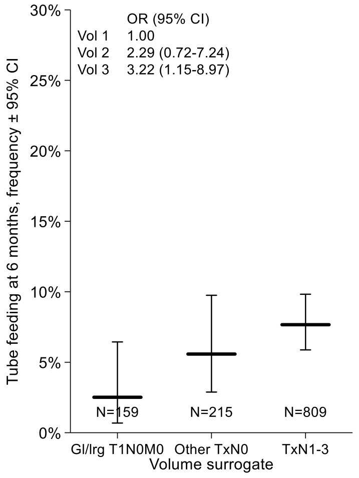

STATacute

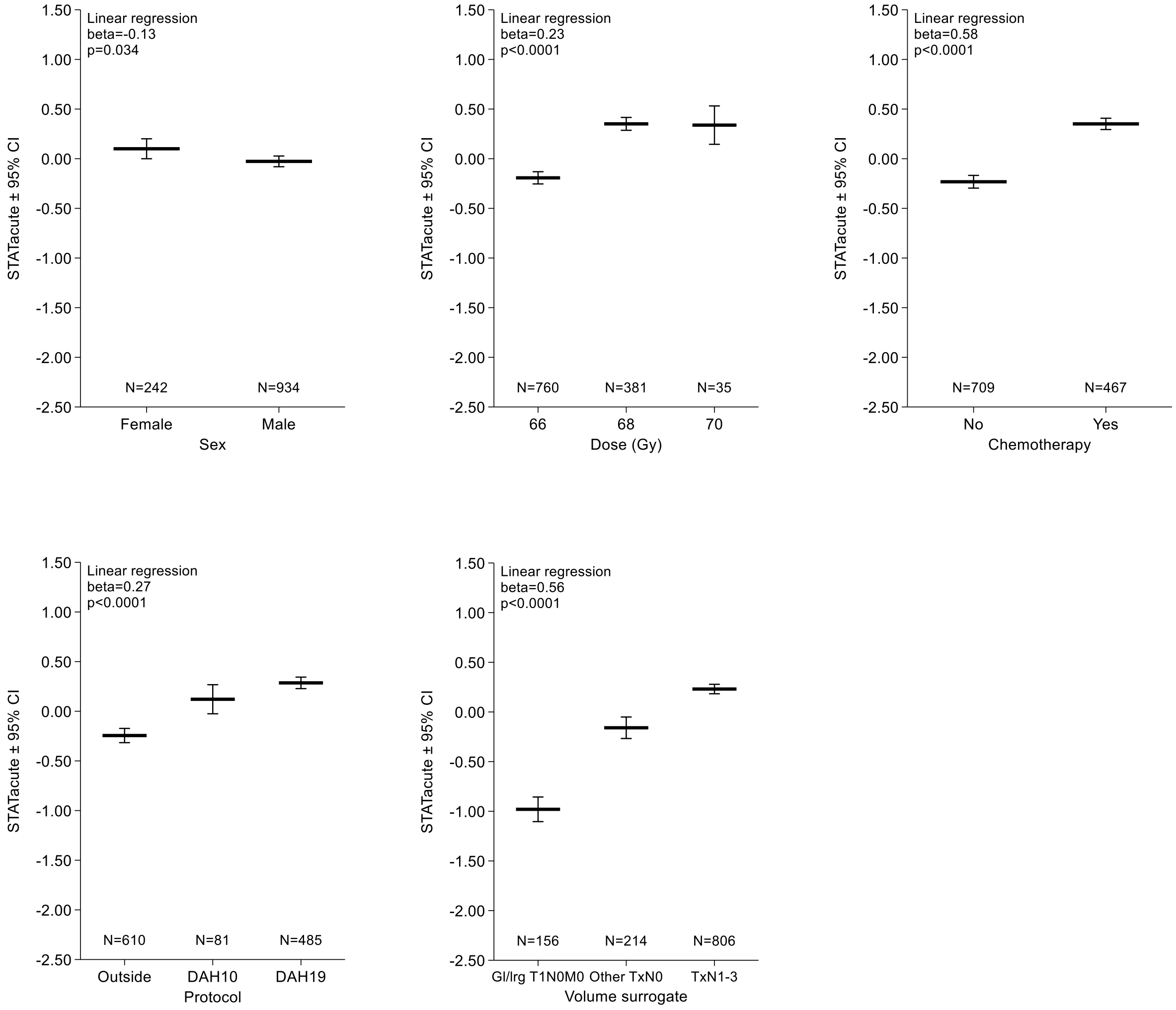

STATlate

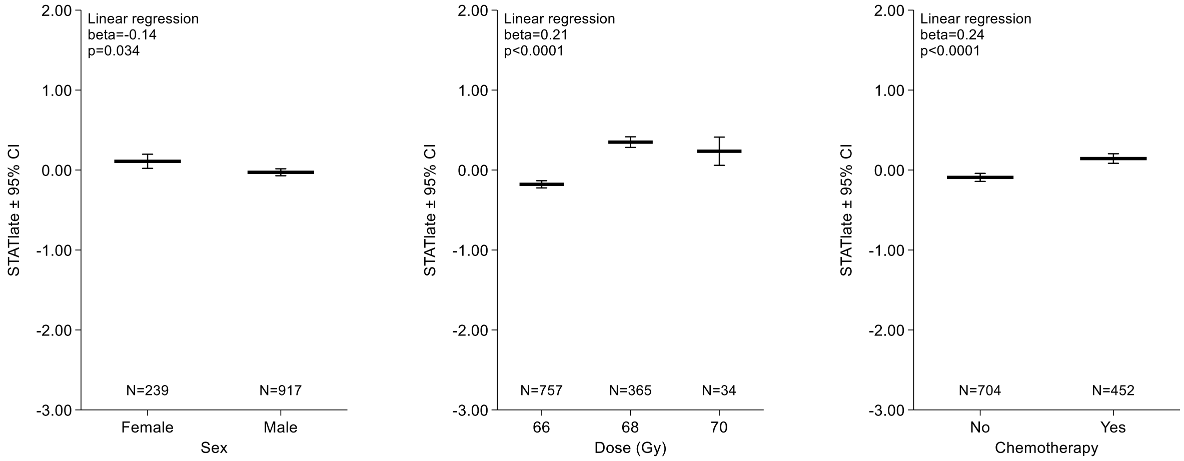

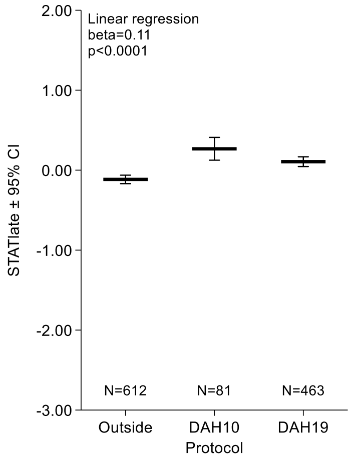
Linear regression
beta=0.11
p<0.0001
STATlate ± 95% CI
2.00
1.00
0.00
-1.00
-2.00
-3.00
N=612
N=81
N=463
Outside
DAH10
DAH19
Protocol

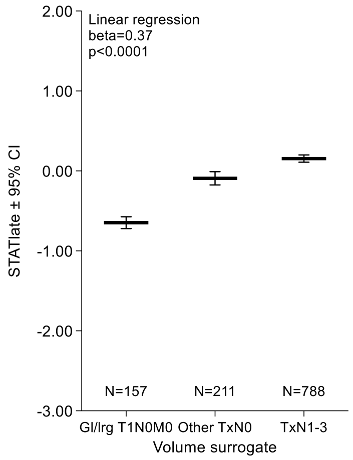
Linear regression
beta=0.37
p<0.0001
STATlate ± 95% CI
2.00
1.00
0.00
-1.00
-2.00
-3.00
N=157
N=211
N=788
Gl/lrg T1N0M0
Other TxN0
TxN1-3
Volume surrogate

STATglobal

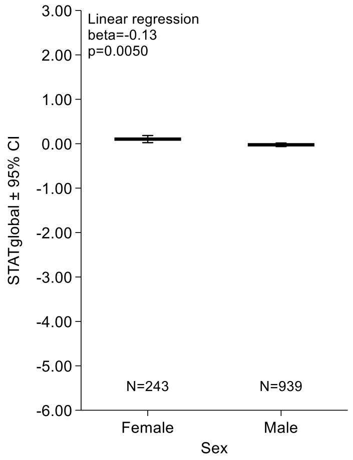
Linear regression
beta=-0.13
p=0.0050
STATglobal ± 95% CI
3.00
2.00
1.00
0.00
-1.00
-2.00
-3.00
-4.00
-5.00
-6.00
N=243
N=939
Female
Male
Sex

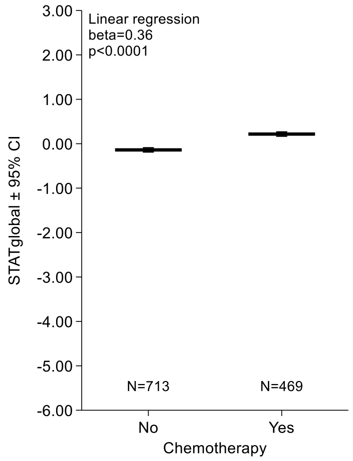
Linear regression
beta=0.36
p<0.0001
STATglobal ± 95% CI
3.00
2.00
1.00
0.00
-1.00
-2.00
-3.00
-4.00
-5.00
-6.00
N=713
N=469
No
Yes
Chemotherapy

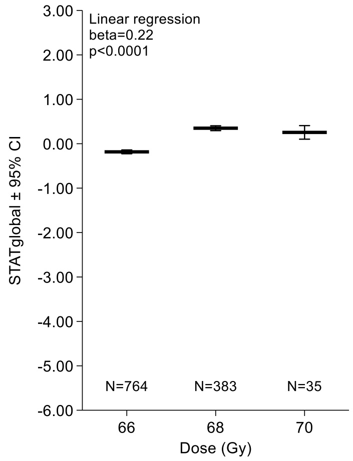
Linear regression
beta=0.22
p<0.0001
STATglobal ± 95% CI
3.00
2.00
1.00
0.00
-1.00
-2.00
-3.00
-4.00
-5.00
-6.00
N=764
N=383
N=35
66
68
70
Dose (Gy)

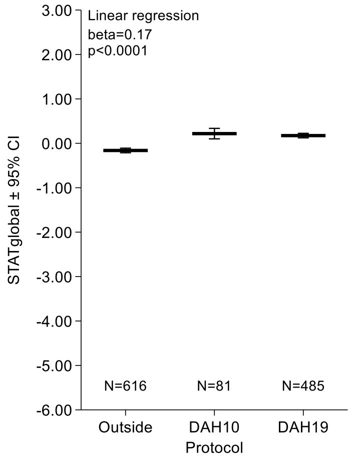
Linear regression
beta=0.17
p<0.0001
STATglobal ± 95% CI
3.00
2.00
1.00
0.00
-1.00
-2.00
-3.00
-4.00
-5.00
-6.00
N=616
N=81
N=485
Outside
DAH10
DAH19
Protocol

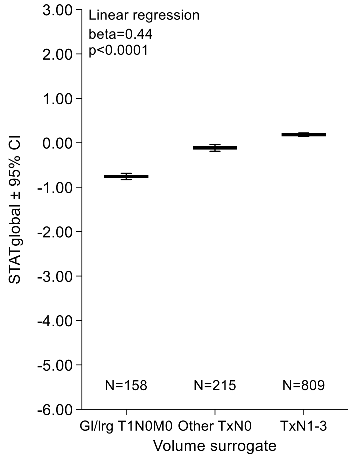
Linear regression
beta=0.44
p<0.0001
STATglobal ± 95% CI
3.00
2.00
1.00
0.00
-1.00
-2.00
-3.00
-4.00
-5.00
-6.00
N=158
N=215
N=809
Gl/lrg T1N0M0
Other TxN0
TxN1-3
Volume surrogate

Supplementary Figure 2. Power analysis for the DAHANCA discovery study. Examples are shown for endpoint frequencies of 0.14 corresponding to severe fibrosis (A), 0.53 corresponding to moderate/severe fibrosis (B), and 0.74 corresponding to moderate/severe mucositis (C).

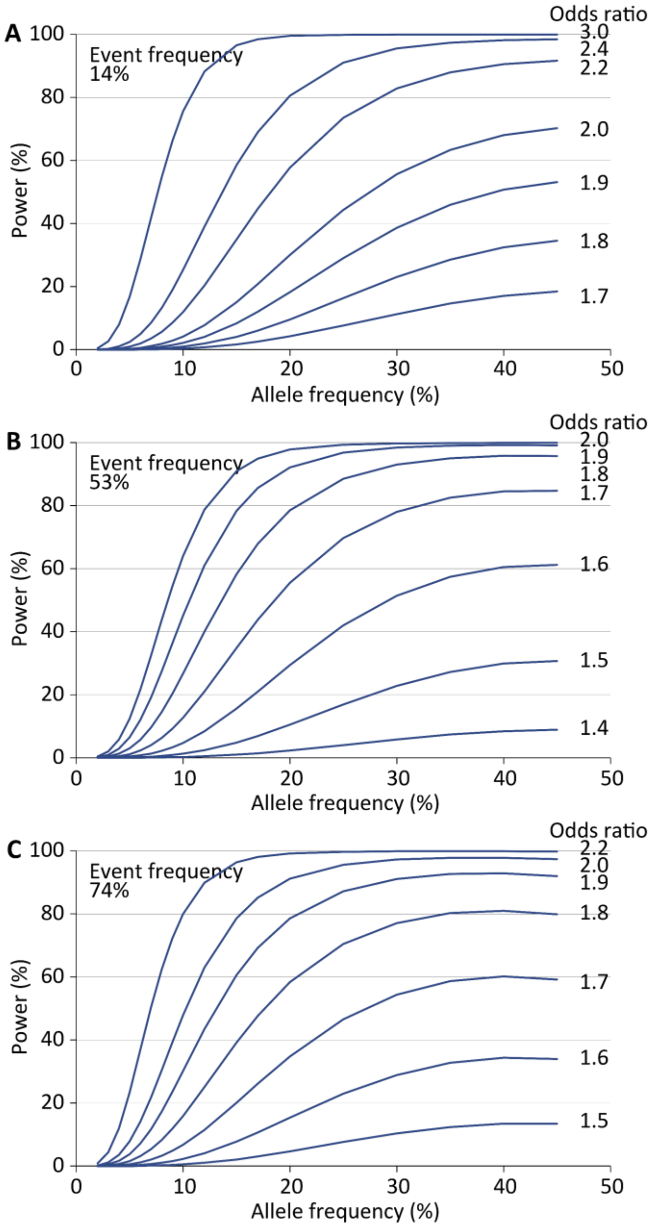

Supplementary Figure 3. QQ and Manhattan plots for the DAHANCA discovery study.

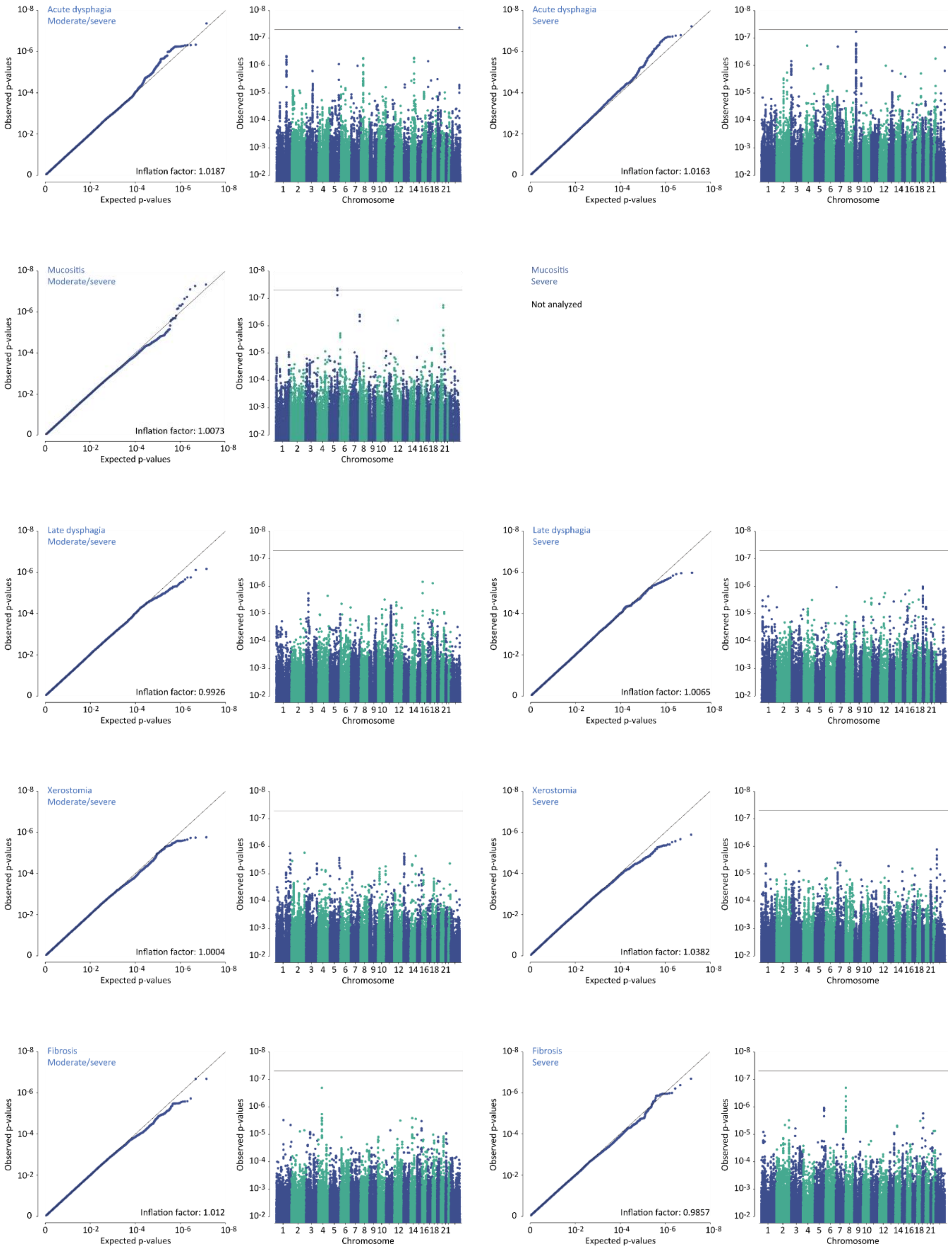

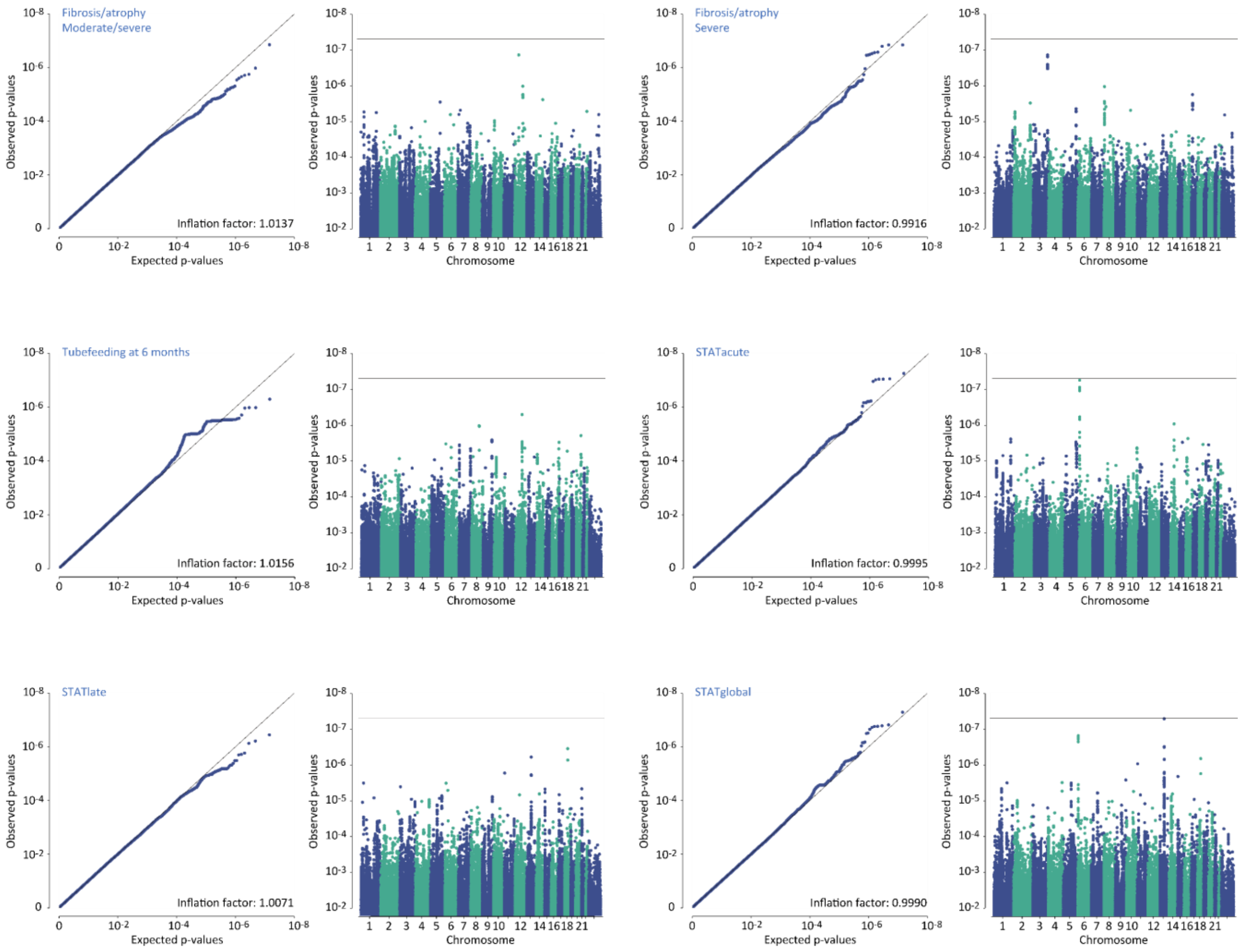
Fibrosis/atrophy
Moderate/severe
Inflation factor: 1.0137
Fibrosis/atrophy
Severe
Inflation factor: 0.9916
Tubefeeding at 6 months
Inflation factor: 1.0156
STATacute
Inflation factor: 0.9995
STATlate
Inflation factor: 1.0071
STATglobal
Inflation factor: 0.9990
Observed p-values
Expected p-values
Chromosome

Supplementary Figure 4. LD plots for loci on chromosome 5 associated with mucositis (A) and on chromosome 6 near-significantly associated with STATacute (B) for the DAHANCA discovery study.

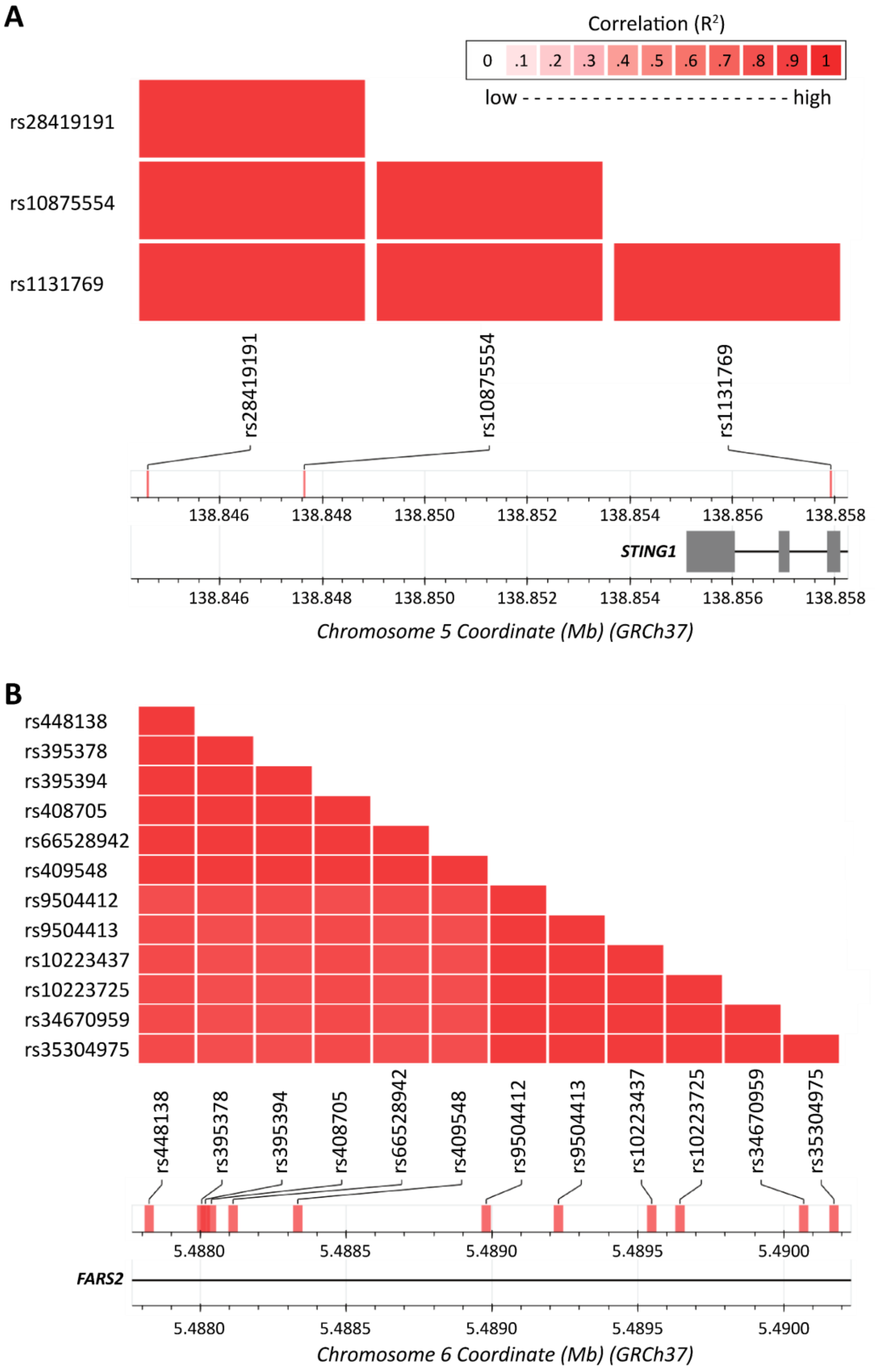

Supplementary Figure 5. Gene expression (TPM, transcripts per million) in various tissue types for genes in the locus on Chr 5 associated with radiation-induced mucositis. Data from The Genotype-Tissue Expression (GTEx) Project, www.gtexportal.org.

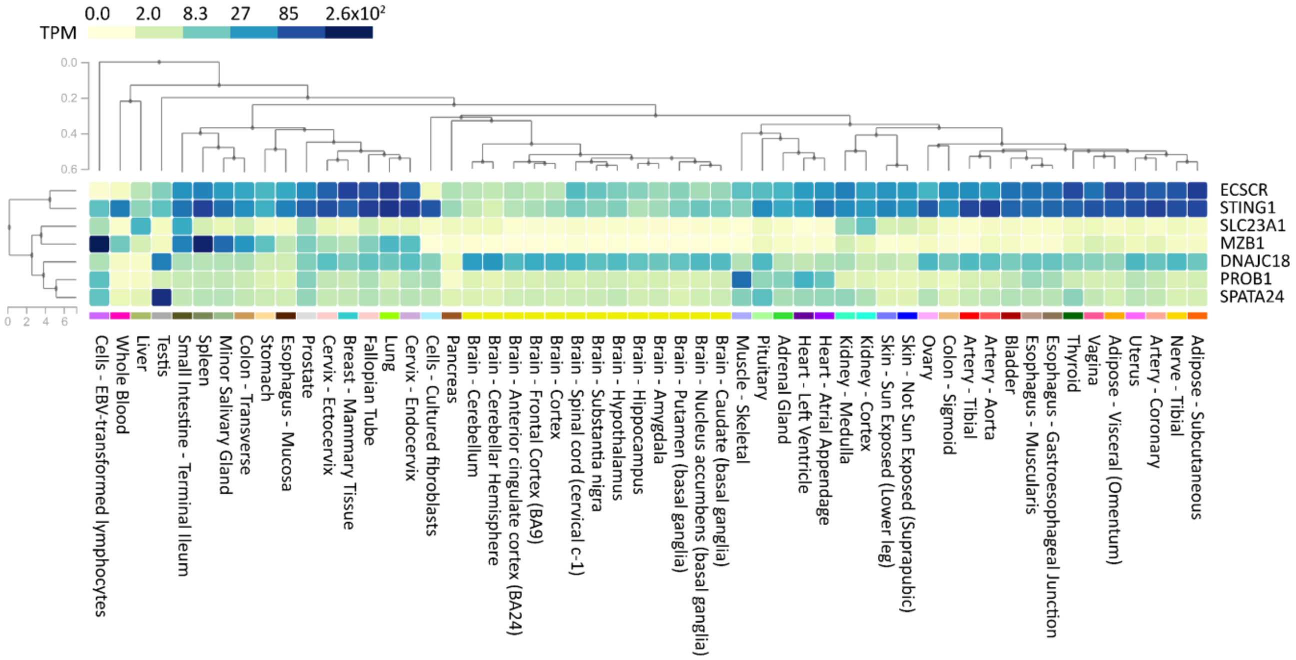

Supplementary Figure 6. Locus Zoom for the DAHANCA discovery study plot for locus on chromosome 6 possibly associated with STATacute with the rs448138*T allele reaching β=0,2437; SE=0,0445; p=5,50·10^{-8} ; MAF=0,18; info=0,99. Variants in orange or red colour are in Linkage Disequilibrium with the specified SNP rs448138. The locus is not in cis-eQTL / sQTL with any of the genes.

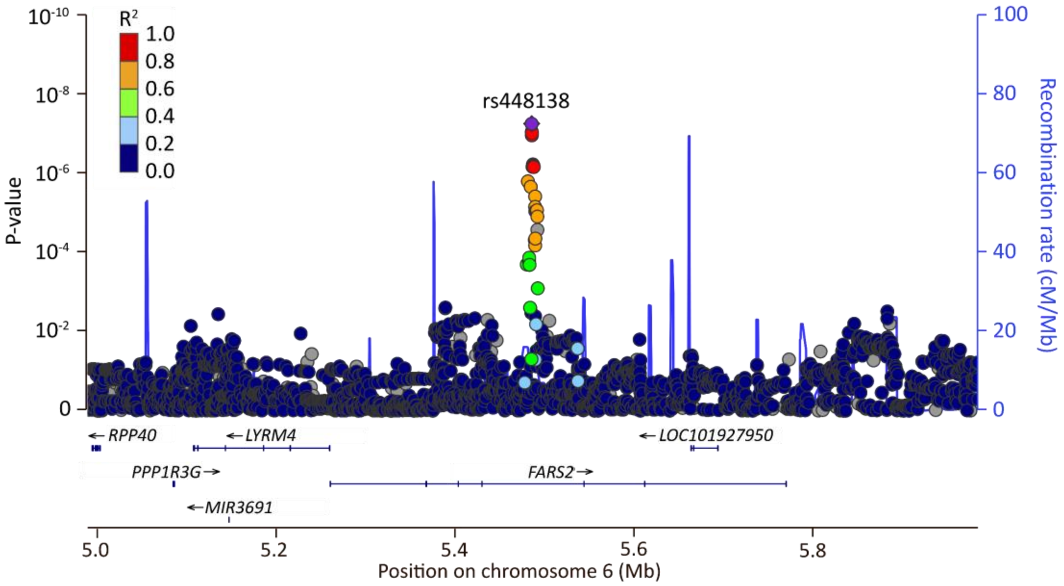

Supplement: Supplementary file 1 — Supplementary Material [file 41416_2021_1670_MOESM1_ESM.pdf]
